# Prenatal Diet and Infant Growth From Birth to Age 24 Months

**DOI:** 10.1001/jamanetworkopen.2024.45771

**Published:** 2024-11-21

**Authors:** Monique M. Hedderson, Holly B. Schuh, Emily A. Knapp, Traci A. Bekelman, Diane J. Catellier, Matt Westlake, Kristen Lyall, Rebecca J. Schmidt, Anne L. Dunlop, Sarah S. Comstock, Leda Chatzi, Katherine A. Sauder, Dana Dabelea, Karen M. Switkowski, Pi-I Debby Lin, Lyndsay A. Avalos, Yeyi Zhu, Assiamira Ferrara

**Affiliations:** 1 Division of Research and Center for Upstream Prevention of Adiposity and Diabetes Mellitus, Kaiser Permanente Northern California, Pleasanton; 2 Department of Epidemiology, Johns Hopkins Bloomberg School of Public Health, Baltimore, Maryland; 3 Lifecourse Epidemiology of Adiposity and Diabetes Center, University of Colorado Anschutz Medical Campus, Aurora; 4 RTI International, Research Triangle Park, North Carolina; 5 A.J. Drexel Autism Institute, Drexel University, Philadelphia, Pennsylvania; 6 Department of Public Health Sciences, University of California, Davis; 7 Department of Gynecology and Obstetrics, Emory University School of Medicine, Atlanta, Georgia; 8 Department of Food Science and Human Nutrition, Michigan State University, East Lansing; 9 Department of Population and Public Health Sciences, University of Southern California, Los Angeles; 10 Department of Implementation Science, Wake Forest University School of Medicine, Winston-Salem, North Carolina; 11 Division of Chronic Disease Research Across the Lifecourse, Department of Population Medicine, Harvard Medical School and Harvard Pilgrim Health Care Institute, Boston, Massachusetts

## Abstract

**Importance:**

Being born either small for gestational age (SGA) or large for gestational age (LGA) and experiencing rapid or slow growth after birth are associated with later-life obesity. Understanding the associations of dietary quality during pregnancy with infant growth may inform obesity prevention strategies.

**Objective:**

To evaluate the associations of prenatal dietary quality according to the Healthy Eating Index (HEI) and the Empirical Dietary Inflammatory Pattern (EDIP) with infant size at birth and infant growth from birth to age 24 months.

**Design, Setting, and Participants:**

This cohort study used data from birthing parent–child dyads in 8 cohorts participating in the Environmental influences on Child Health Outcomes program between 2007 and 2021. Data were analyzed from March 2021 to August 2024.

**Exposures:**

The HEI and the EDIP dietary patterns.

**Main Outcomes and Measures:**

Outcomes of interest were infant birth weight, categorized as SGA, reference range, or LGA, and infant growth from birth to ages 6, 12, and 24 months, categorized as slow growth (weight-for-length *z* score [WLZ] score difference <−0.67), within reference range (WLZ score difference −0.67 to 0.67), or rapid (WLZ score difference, >0.67).

**Results:**

The study included 2854 birthing parent–child dyads (median [IQR] maternal age, 30 [25-34] years; 1464 [51.3%] male infants). The cohort was racially and ethnically diverse, including 225 Asian or Pacific Islander infants (7.9%), 640 Black infants (22.4%), 1022 Hispanic infants (35.8%), 664 White infants (23.3%), and 224 infants (7.8%) with other race or multiple races. A high HEI score (>80), indicative of a healthier diet, was associated with lower odds of LGA (adjusted odds ratio [aOR], 0.88 [95% CI, 0.79-0.98]), rapid growth from birth to age 6 months (aOR, 0.80 [95% CI, 0.37-0.94]) and age 24 months (aOR 0.82 [95% CI, 0.70- 0.96]), and slow growth from birth to age 6 months (aOR, 0.65 [95% CI, 0.50-0.84]), 12 months (aOR, 0.74 [95% CI, 0.65-0.83]), and 24 months (OR, 0.65 [95% CI, 0.56-0.76]) compared with an HEI score 80 or lower. There was no association between high HEI and SGA (aOR, 1.14 [95% CI, 0.95-1.35]). A low EDIP score (ie, ≤63.6), indicative of a less inflammatory diet, was associated with higher odds of LGA (aOR, 1.24 [95% CI, 1.13-1.36]) and rapid infant growth from birth to age 12 months (aOR, 1.50 [95% CI, 1.18-1.91]) and lower odds of rapid growth to age 6 months (aOR, 0.77 [95% CI, 0.71-0.83]), but there was no association with SGA (aOR, 0.80 [95% CI, 0.51-1.25]) compared with an EDIP score of 63.6 or greater.

**Conclusions and Relevance:**

In this cohort study, a prenatal diet that aligned with the US Dietary Guidelines was associated with reduced patterns of rapid and slow infant growth, known risk factors associated with obesity. Future research should examine whether interventions to improve prenatal diet are also beneficial in improving growth trajectory in children.

## Key Points

**Question:**

Is prenatal dietary quality, assessed with the healthy eating index (HEI) and the empirical dietary inflammatory pattern (EDIP), associated with infant size at birth and infant growth from birth to age 24 months?

**Findings:**

In this cohort study of 2854 birthing parent–child dyads, higher prenatal HEI scores (indicating healthier diet) were associated with birth weight and growth kinetics within reference ranges. The associations between EDIP and infant growth were less clear.

**Meaning:**

These findings suggest that a healthy prenatal diet may reduce patterns of infant growth outside reference ranges and may represent an upstream obesity prevention strategy.

## Teaser

This cohort study evaluates the associations of prenatal dietary quality according to the Healthy Eating Index, based on the US Dietary Guidelines, and the Empirical Dietary Inflammatory Pattern with infant size at birth and infant growth from birth to age 24 months

## Introduction

Almost one-third of children in the US have overweight or obesity.^1^ Given the adverse cardiometabolic health effects associated with obesity, several government and medical institutes have urged for more research to inform the upstream prevention of obesity.^2^ Nutrition during pregnancy, a sensitive developmental window, may be associated with both infant size at birth and longer-term growth kinetics.^3,4,5,6,7,8,9,10^ Being born large for gestational age (LGA) is associated with later-life obesity,^11,12^ whereas being born small for gestational age (SGA) is associated with several adverse cardiometabolic outcomes.^13^ Both slow and rapid weight gain during the first year of life are associated with later-life obesity risk.^14^ However, most previous studies on prenatal diet examined birth weight and lacked information on infant growth.^7,15,16,17^ Many prior studies of pregnancy diet and infant growth focused on the intake of single foods in isolation^7,15,16,17^; however, dietary indices better account for the combined effects of foods and nutrients.

Two dietary indices defined a priori are the 2015 Healthy Eating Index (HEI), a measure of healthy diet based on adherence to the US Department of Agriculture (USDA) Dietary Guidelines for Americans,^18^ and the Empirical Dietary Inflammatory Pattern (EDIP),^19^ an index developed to reflect the proinflammatory potential of the diet, which has been associated with levels of inflammatory markers and cardiometabolic outcomes outside of pregnancy.^20^ A recent meta-analysis found that a higher maternal dietary inflammatory index score was associated with an increased risk of SGA and low birth weight infants but no significant association with later-life obesity.^21^ However, there is limited research on whether dietary patterns, such as the HEI or the EDIP, during pregnancy are associated with infant growth during the first 2 years of life.

The overall aim of this prospective study was to investigate the association between prenatal diet according to the HEI or EDIP with infant size at birth as well as with rapid and slow infant growth between birth and ages 6, 12, and 24 months. We used data from the Environmental influences on Child Health Outcomes (ECHO) consortium to test the hypothesis that adherence to a more healthful (ie, higher HEI) or less inflammatory (ie, lower EDIP) dietary pattern during pregnancy will be associated with a healthier infant growth pattern from birth to age 24 months.

## Methods

For this cohort study, the institutional review boards of record for each study site, as well as the host institution of the ECHO Data Analysis Center (Johns Hopkins Bloomberg School of Public Health), approved all activities associated with this study. All participants provided written informed consent. This study followed the Strengthening the Reporting of Observational Studies in Epidemiology (STROBE) reporting guideline for cohort studies.

### Study Population

The ECHO consortium combines longitudinal data across ongoing child cohort studies throughout the US that are now collecting data under a common protocol, with the aim of achieving demographic and geographic diversity and large sample sizes to address research questions pertaining to environmental exposures and child health outcomes.^22,23^ Children from the ECHO consortium who met the following criteria were included in our analyses: singleton births with at least 32 weeks’ gestation at birth, with weight and length measured at birth and at least 1 other time point (age 6, 12, or 24 months), and whose birthing parent has prenatal dietary data. Infants whose data are included in this study were born between 2007 and 2021 (from 8 cohorts and 9 sites) (eFigure 1 in Supplement 1). All cohorts included in this analysis recruited pregnant participants during pregnancy. eTable 1 in Supplement 1 compares children included in this analytic sample with the 7007 children excluded who had anthropometric measures but no prenatal diet data.

### Outcomes

Anthropometric measures included both medical record abstraction (52.6% of birth measurements; 60.5% of measurements from ages 6, 12, and 24 months) and study measures (47.4% of birth measurements; 39.5% of study measures). All recumbent length and weight measurements occurring between ages 0 and 24 months of age were used to calculate weight-for-length *z* scores (WLZ) using the World Health Organization macros.^24,25^ Weight at birth was categorized as SGA, reference range, or LGA using Intergrowth-21st growth percentiles for gestational age and sex.^26^ Each child’s measurements were binned into 6-, 12-, and 24-month age categories. All age bins included 1 month before and after the age of interest (eg, age 6 months measurements included measurements occurring within 30 days before or after age 180 days). We created 3 ordinal variables for 6-, 12-, and 24-month measurements, comparing WLZ scores at any of the 3 ages with the child’s birth WLZ score to measure an absolute change between the paired measurements. We used 3 since-birth growth categories: reference range (WLZ score difference, −0.67 to 0.67), rapid (WLZ score difference, >0.67), and slow growth (WLZ score difference, <−0.67).^11,12^ Because extremely rapid infant weight gain is associated with a particularly high risk of overweight and obesity,^27^ a second set of the 3 pairwise, ordinal variables was derived using the same 3 growth categories with the addition of 2 categories for extreme rapid growth (WLZ score difference, >1.34) and extreme slow growth (WLZ score difference, <−1.34). We examined both outcome variables and their associations with the primary exposures.

### Exposures

Primary exposures of interest were measures of prenatal diet quality. Analysts at the centralized ECHO data analysis center^28^ harmonized dietary data across cohorts and assessment methods (food frequency questionnaire [FFQ] vs 24-hour recalls) and calculated scores from dietary intake data provided by cohorts.^29^ Details of dietary intake type and timing across cohorts are provided in eTable 2 in Supplement 1. If more than 1 diet assessment was available, we selected the earliest prenatal dietary assessment per participant. Prenatal dietary data were used to derive 2 measures of diet quality: the 2015 HEI^30,31^ and the EDIP scores. The HEI ranges from 0 to 100, with a score of 100 indicating a diet that meets the recommendations from the 2015 to 2020 Dietary Guidelines for Americans. HEI diet scores were dichotomized according to high quality (HEI >80) vs low quality based on the USDA current recommendations.^32^ For a subset of our cohort with data available, we calculated the Alternate Healthy Eating Index for Pregnancy (AHEI-P), a validated measure that assesses overall dietary quality in relation to the 2010 USDA Dietary Guidelines for Americans, modified for pregnancy.^33^ No standard cutoff exists for AHEI-P, so the distribution of AHEI-P scores was used to determine a cutoff, with a healthy diet being defined as a score greater than 76.3, the mean value below the fourth quartile. The EDIP is only validated for use with FFQ data and was not calculated for the 24-hour recall cohorts.^19^ It has 18 components and is calculated by summing the weighted mean daily intake for each component. Nine components are considered anti-inflammatory, including beer, wine, tea, coffee, dark yellow vegetables, leafy green vegetables, snacks, fruit juice, and pizza, and 9 are considered inflammatory, including processed meat, red meat, organ meat, fish (other than dark-meat fish), other vegetables, refined grains, high-energy beverages, low-energy beverages, and tomatoes. Because no standard cutoff exists for EDIP, the distribution of EDIP scores was used to determine a cutoff, with a less inflammatory EDIP score defined as 63.6 or less, the mean value below the fourth quartile, for distinguishing high-quality (less inflammatory) vs low-quality (more inflammatory) diet. We also evaluated the associations by quartiles of EDIP.

### Covariates

Covariates of interest included maternal education (categorical), child sex at birth (dichotomous), maternal age at delivery (categorical), prepregnancy body mass index (BMI; calculated as weight in kilograms divided by height in meters squared) (categorical), prenatal tobacco use (dichotomous), prenatal diet data source (24-hour recall or FFQ), trimester of prenatal diet collection (categorical), and size for gestational age (categorical, only adjusted for when examining infant growth outcomes). Child race (Asian, Black, Native Hawaiian or Other Pacific Islander, White, multiple races, or other race [ie, participants did not identify any of the listed categories and provided free-text answers]) and Hispanic or non-Hispanic ethnicity were obtained from maternal or caregiver reports or medical records. Due to the small sample size, we combined children whose race were American Indian or Alaska Native into the other race group and children who were Native Hawaiian or Other Pacific Islander into the Asian race group and children of multiple races or other racial groups into single categories. We viewed race and ethnicity as social constructs rather than biological causes of disease risk^34^ and they were evaluated as a covariate in this study given known racial and ethnic differences in both pregnancy diet^35^ and child growth.^36^ Due to high missingness (>65%), variables for infant feeding practices could not be included in the analysis.

### Statistical Analysis

Data from all participating cohorts were pooled prior to analysis. We examined distributions of participant characteristics by dietary data source (FFQ vs 24-hour recalls) using χ^2^ for comparison of proportions and Wilcoxon rank-sum for nonparametric data. We analyzed data using multinomial logistic regression models and calculated adjusted standard errors by cohort to account for correlated observations between children in the same cohort using a clustered sandwich estimator. Individual-level covariates had low (<5%) missingness when evaluated, and these observations were excluded from the analysis. Adjusted odds ratios (aORs) were calculated using 3 age-specific (growth since birth at 6, 12, and 24 months) multivariable models that included adjustment for the covariates of interest.

*P* values were 2-sided, and statistical significance was set at α = .05. All analyses were performed using Stata version 17 (StataCorp). Data were analyzed from March 2021 to August 2024.

## Results

Of 60 045 birthing parent–child pairs in ECHO, 48 684 were singleton births. After 1802 very-preterm (<32 weeks’ gestation) births were excluded, 10 667 children had a WLZ measure at birth and at least 1 measure between birth and age 6, 12, or 24 months. Among these, prenatal dietary data were available for 2957 of the birthing parent–child pairs, and EDIP or HEI diet quality scores could be derived for 2854 birthing parent–child pairs (median [IQR] maternal age, 30 [25-34] years; 1464 [51.3%] male infants), constituting our study sample (Table 1; eFigure 1 in Supplement 1).

**Table 1.** Characteristics of Study Sample Overall and by Dietary Assessment Tool Among Birthing Parent–Child Pairs in the ECHO Program

Characteristic	Participants, No. (%)^a^		
	Total (N = 2854)	Dietary assessment tool	
		24-h Recall (n = 549)	Food frequency questionnaire (n = 2305)
**Child characteristics**			
Birth year			
2007-2011	952 (33.4)	112 (20.4)	840 (36.4)
2012-2016	1275 (44.7)	184 (33.5)	1091 (47.3)
2017-2021	627 (22.0)	253 (46.1)	374 (16.2)
Child sex at birth			
Female	1390 (48.7)	277 (50.5)	1113 (48.3)
Male	1464 (51.3)	272 (49.5)	1192 (51.7)
Child race and ethnicity^b^			
Asian, Native Hawaiian, or Other Pacific Islander	225 (7.9)	5 (0.9)	220 (9.5)
Black	640 (22.4)	<60	<590
Hispanic	1022 (35.8)	289 (52.6)	734 (31.8)
White	664 (23.3)	160 (29.1)	504 (21.9)
Other or multiracial	224 (7.8)	37 (6.7)	187 (8.1)
Missing	79 (2.8)	<5	<79
Birth weight, g	3325 (3040-3650)	3270 (2990-3580)	3340 (3050-3650)
Size at birth			
Reference range	2294 (80.4)	449 (81.8)	1845 (80.0)
Small for gestational age	155 (5.4)	<30	<130
Large for gestational age	404 (14.2)	71 (12.9)	333 (14.4)
Missing	<5	0	<5
Preterm birth	138 (4.8)	33 (6.0)	105 (4.6)
**Maternal characteristics**			
Age at delivery, y			
<25	586 (20.5)	126 (23.0)	460 (20.0)
25-29	763 (26.7)	139 (25.3)	624 (27.1)
30-34	917 (32.1)	174 (31.7)	743 (32.2)
≥35	588 (20.6)	110 (20.0)	478 (20.7)
Prepregnancy BMI			
18.5-24.9	1156 (40.5)	218 (39.7)	938 (40.7)
<18.5	74 (2.6)	12 (2.2)	62 (2.7)
25-29.9	767 (26.9)	157 (28.6)	610 (26.5)
≥30	844 (29.6)	155 (28.2)	689 (29.9)
Missing	13 (0.5)	7 (1.3)	6 (0.3)
Educational status, highest			
<College degree	1562 (54.7)	341 (62.1)	1221 (53.0)
College degree	744 (26.1)	107 (19.5)	637 (27.6)
Graduate degree	501 (17.6)	81 (14.8)	420 (18.2)
Missing	47 (1.6)	20 (3.6)	27 (1.2)
Income during pregnancy, $			
<30 000	643 (22.5)	135 (24.6)	508 (22.0)
30 000-49 999	219 (7.7)	34 (6.2)	185 (8.0)
50 000-74 999	257 (9.0)	<5	<257
75 000-99 999	171 (6.0)	<5	<171
≥100 000	550 (19.3)	26 (4.7)	524 (22.7)
Missing	1014 (35.5)	352 (64.1)	662 (28.7)
Prenatal tobacco or nicotine use			
Yes	135 (4.7)	22 (4.0)	113 (4.9)
Missing	<5	<5	<5
Timing of dietary assessment, trimester			
First	934 (32.7)	48 (8.7)	886 (38.4)
Second	1458 (51.1)	245 (44.6)	1213 (52.6)
Third	450 (15.8)	<260	<200
Unknown	12 (0.4)	<5	<12
High HEI (healthy)^c^			
No	2551 (89.4)	547 (99.6)	2004 (86.9)
Yes	303 (10.6)	<5	<303
Low EDIP (less inflammatory)^d^			
No	2074 (72.7)	NA	2074 (90.0)
Yes	205 (7.2)	NA	205 (8.9)
Missing	575 (20.2)	549 (100)	26 (1.1)

Abbreviations: BMI, body mass index (calculated as weight in kilograms divided by height in meters squared); EDIP, Empirical Dietary Inflammatory Pattern; HEI, Healthy Eating Index; NA, not applicable.

^a^ In accordance with publication and data use policy from the Environmental influences on Child Health Outcomes program, cell sizes smaller than 5 are suppressed for privacy. The sample size in an additional column is also suppressed to prevent calculation of the exact sample size in cells with fewer than 5 participants.

^b^ The Hispanic ethnicity category includes children of all races, while other racial categories only include non-Hispanic children. The other or multiracial category includes parent report of child race as American Indian or Alaska Native (6 infants), multiple race (179 infants), and participants who did not identify any of the listed categories and provided free-text answers (39 infants).

^c^ High HEI was defined as a score greater than 80. The HEI ranges from 0 to 100, with a higher score indicating greater adherence to the Dietary Guidelines for Americans.

^d^ Low EDIP is defined as a score of 63.6% or lower, using the lowest quartile-based cutoff, indicating a less inflammatory diet pattern.

There were 1156 birthing parents (40.5%) with BMI between 18.5 and 24.6 before pregnancy, and 135 birthing parents (4.7%) used tobacco during pregnancy (Table 1). Approximately one-quarter of birthing parents (744 [26.1%]) had a college degree and 501 (17.5%) had a graduate degree. The cohort was racially and ethnically diverse, including 225 Asian or Pacific Islander infants (7.9%), 640 Black infants (22.4%), 1022 Hispanic infants (35.8%), and 664 White infants (23.3%), and 224 infants (7.8%) were other race or multiple races (Table 1).

FFQs were the most common dietary assessment used (2305 participants [80.8%]), and 886 FFQs (38.4%) were administered in the first trimester of pregnancy. A total of 303 participants (10.6%) had HEI scores in the high category (>80, indicating a healthier diet), while 205 participants (8.9%) had low EDIP values (≤63.6, indicating a less inflammatory diet) (Table 1). From birth to ages 6, 12, and 24 months, 30%, 39%, and 23% of children experienced rapid growth, and 15%, 9%, and 7.5% experienced slow growth, respectively (Table 2). Pregnancies with high HEI scores had a higher percentage with growth within reference ranges at birth, 6, 12, and 24 months (eTable 1 in Supplement 1). Pregnancies with a low EDIP score had a higher percentage of LGA infants and slow growth at 12 and 24 months but more growth within reference range at 6 months (eTable 2 in Supplement 1).

**Table 2.** Infant Size at Birth and Child Growth Outcomes, Overall and by Dietary Assessment Tool

Outcome	Infants, No. (%)^a^		
	Total (N = 2854)	Dietary assessment tool	
		24 h recall (n = 549)	Food frequency questionnaire (n = 2305)
**Size at birth**			
Reference range	2294 (80.4)	449 (81.8)	1845 (80.0)
Small for gestational age	155 (5.4)	<30	<130
Large for gestational age	404 (14.2)	71 (12.9)	333 (14.4)
Missing	<5	0	<5
**Growth (change in WLZ) from birth**			
Age 6 mo			
Reference range (−0.67 to 0.67)	609 (21.3)	163 (29.7)	446 (19.3)
Rapid growth (>0.67)	867 (30.4)	214 (39.0)	653 (28.3)
Slow growth (<−0.67)	416 (14.6)	154 (28.1)	262 (11.4)
Missing	962 (33.7)	18 (3.3)	944 (41.0)
Age 12 mo			
Reference range (−0.67 to 0.67)	487 (17.1)	45 (8.2)	442 (19.2)
Rapid growth (>0.67)	1110 (38.9)	91 (16.6)	1019 (44.2)
Slow growth (<−0.67)	251 (8.8)	20 (3.6)	231 (10.0)
Missing	1006 (35.2)	393 (71.6)	613 (26.6)
Age 24 mo			
Reference range (−0.67 to 0.67)	339 (11.9)	<5	<339
Rapid growth (>0.67)	652 (22.8)	15 (2.7)	637 (27.6)
Slow growth (−0.67)	214 (7.5)	<5	<214
Missing	1650 (57.8)	530 (96.5)	1120 (48.6)
**Growth (change in WHZ) from birth** ^b^			
Age 6 mo			
Reference range (−0.67 to 0.67)	607 (21.4)	162 (29.6)	445 (19.4)
Rapid growth (≥0.67)	323 (11.4)	93 (17.0)	230 (10.0)
Slow growth (≤−0.67)	186 (6.5)	61 (11.1)	125 (5.4)
Extreme rapid growth (>1.34)^e^	540 (19.0)	121 (22.1)	419 (18.3)
Extreme slow growth (<−1.34)^f^	228 (8.0)	93 (17.0)	135 (5.9)
Missing	958 (33.7)	18 (3.3)	940 (41.0)
Age 12 mo			
Reference range (−0.67 to 0.67)	487 (17.1)	45 (8.2)	442 (19.3)
Rapid growth (≥0.67)	324 (11.4)	32 (5.8)	292 (12.7)
Slow growth (≤-0.67)	146 (5.1)	7 (1.3)	139 (6.1)
Extreme rapid growth (>1.34)	783 (27.6)	59 (10.8)	724 (31.6)
Extreme slow growth (<-1.34)	104 (3.7)	13 (2.4)	91 (4.0)
Missing	998 (35.1)	392 (71.5)	606 (26.4)
Age 24 mo			
Reference range (−0.67 to 0.67)	<340	<5	334 (14.6)
Rapid growth (≥0.67)	<180	<5	173 (7.5)
Slow growth (≤−0.67)	<105	<5	99 (4.3)
Extreme rapid growth (>1.34)	472 (16.6)	12 (2.2)	460 (20.1)
Extreme slow growth (<−1.34)	113 (4.0)	0 (0.0)	113 (4.9)
Missing	1644 (57.8)	529 (96.5)	1115 (48.6)

Abbreviation: WHZ, weight-for-height *z* score; WLZ, weight-for-length *z* score.

^a^ In accordance with publication and data use policy from the Environmental influences on Child Health Outcomes program, cell sizes smaller than 5 are suppressed for privacy. The sample size in an additional column is also suppressed to prevent calculation of the exact sample size in cells with fewer than 5 participants.

^d^ Sample sizes were 2842 for the total cohort, 548 for 24-hour recall, and 2294 for the food frequency questionnaire.

^e^ Extreme rapid growth is defined as a difference in WLZ between birth and ages 6, 12, or 24 months of greater than 1.34.

^f^ Extreme slow growth is defined as a difference in WLZ between birth and ages 6, 12, or 24 months of less than −1.34.

Infants with birthing parents with high prenatal HEI score had 12% lower odds of being born LGA (aOR, 0.88 [95% CI, 0.79-0.98]), whereas they did not have statistically significantly increased odds of being born SGA (aOR, 1.14 [95% CI, 0.95-1.35]) compared with infants with birthing parents with a low HEI score (Table 3). Infants born to birthing parents with a low EDIP had increased odds of being born LGA (aOR, 1.24 [95% CI, 1.13-1.36]) compared with infants born to birthing parents with a high EDIP, but EDIP scores were not associated with being born SGA (aOR, 0.80 [95% CI, 0.51-1.24]) (Table 3). There were no clear trends or associations of quartiles of EDIP or high AHEI-P score (>76.3%) with infant size for gestational age (eTable 3 and eTable 4 in Supplement 1).

**Table 3.** Association Between Prenatal Dietary Pattern and Size for Gestational Age at Birth Category

Size for gestational age	Adjusted odds ratio (95% CI)^a^	
	High HEI (n = 2711)^b^	Low EDIP (n = 2159)^c^
Reference range	1 [Reference]	1 [Reference]
Small^d^	1.14 (0.95-1.35)	0.80 (0.51-1.24)
Large^e^	0.88 (0.79-0.98)	1.24 (1.13-1.36)

Abbreviations: EDIP, Empirical Dietary Inflammatory Pattern; HEI, Healthy Eating Index.

^a^ Multinomial logistic regression models adjusted for maternal education, child sex, maternal age at delivery, prepregnancy body mass index, child race and ethnicity, prenatal tobacco use, dietary assessment (food frequency questionnaire or 24-hour recall), and trimester of dietary assessment. Robust standard errors clustered on cohort were included in the estimate to account for correlation of children within the same cohort.

^b^ High HEI was defined as a score greater than 80. The HEI ranges from 0 to 100, with higher score indicating closer adherence to the recommendations from the Dietary Guidelines for Americans.

^c^ Low EDIP was defined as a score of 63.6% or lower, using the lowest quartile-based cutoff, indicating a less inflammatory diet pattern.

^d^ Using Intergrowth-21st reference less than and not including 10th percentile.

^e^ Using Intergrowth-21st reference greater than and not including 90th percentile.

**Figure 1.**
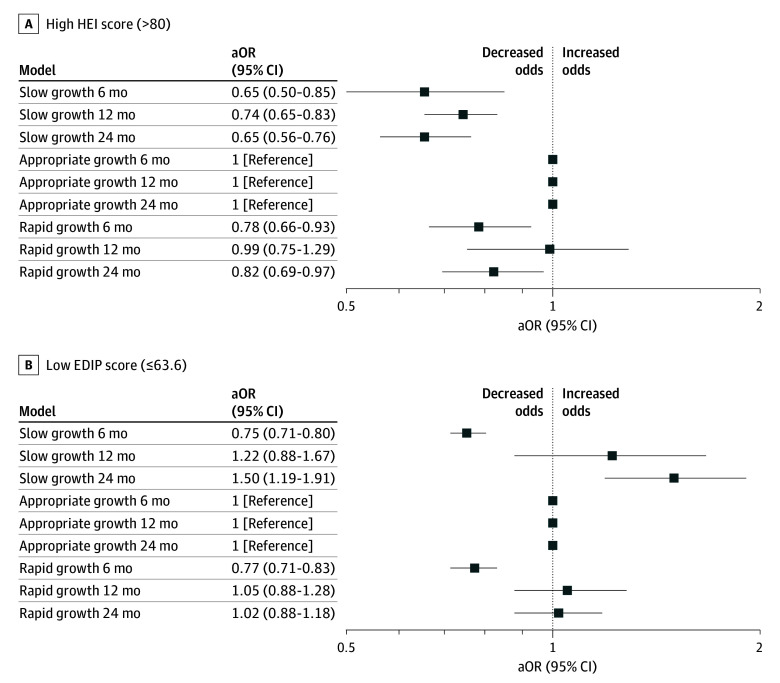
Adjusted Odds Ratios (aORs) of Dietary Patterns and Infant Growth at Ages 6, 12, and 24 Months

**Figure 2.**
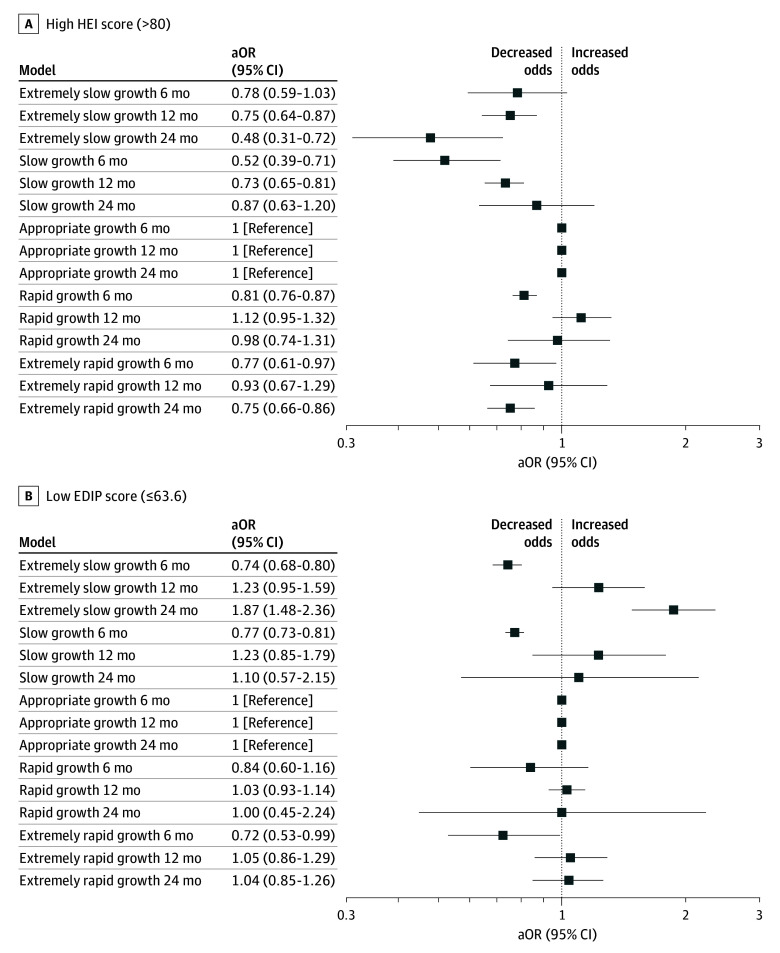
Adjusted Odds Ratios (aORs) of Dietary Patterns and Extremes of Infant Growth at Ages 6, 12, and 24 Months

The infants with birthing parents with high HEI scores during pregnancy had decreased odds of rapid growth between birth and age 6 months (aOR, 0.80 [95% CI, 0.37-0.94]) and between birth and age 24 months (aOR, 0.82 [95% CI, 0.70-0.96]) and decreased odds of slow growth between birth and ages 6 months (aOR, 0.65 [95% CI, 0.51-0.84]), 12 months (aOR, 0.74 [95% CI, 0.65-0.83]), and 24 months (aOR, 0.65 [95% CI, 0.56-0.76]) (Figure 1A). High HEI score was also associated with lower odds of extreme slow growth between birth and ages 12 months (aOR, 0.75 [95% CI, 0.64-0.87]) and 24 months (aOR, 0.48 [95% CI, 0.31, 0.72]) and with lower odds of extreme rapid growth between birth and age 6 months (aOR, 0.77 [95% CI, 0.61-0.97]) and 24 months (aOR, 0.75 [95% CI, 0.66-0.86]) (Figure 2A).

Infants with birthing parents with a low EDIP score had decreased odds of rapid growth (aOR, 0.77 [95% CI, 0.71-0.83]) from birth to 6 months. A low maternal EDIP score during pregnancy was not associated with rapid growth from birth to ages 12 or 24 months but was associated with increased odds of slow growth at age 24 months (aOR, 1.50 [95% CI, 1.18-1.91]) (Figure 1B). Compared with the highest quartile for EDIP (most inflammatory) the 2 lowest quartiles for EDIP were associated with decreased risk of slow and rapid growth at age 6 months and increased risk of slow growth at age 12 months (eTable 4 in Supplement 1). A low maternal EDIP score was associated with lower odds of extreme rapid growth (aOR, 0.72 [95% CI, 0.53-0.99]) and extreme slow growth (aOR, 0.74, [95% CI, 0.68-0.80]) between birth and age 6 months but with increased odds of extreme slow growth from birth to age 24 months (aOR, 1.87 [95% CI, 1.48-2.36]) (Figure 2B).

The highest quartile for prenatal AHEI-P was associated with increased risk of slow growth at age 12 months (aOR, 1.22 [95% CI, 1.17-1.26]) but decreased risk of slow growth at age 24 months (aOR, 0.58 [95% CI, 0.47-0.71]) and no association with rapid growth (eFigure 2 in Supplement 1). The highest quartile of prenatal AHEI-P was associated with decreased risk of extreme slow growth (aOR, 0.58 [95% CI, 0.33-0.99]) but not extreme rapid growth (aOR, 0.86 [95% CI, 0.73-1.00]) at age 24 months (eFigure 3 in Supplement 1).

## Discussion

In this large, racially and ethnically diverse cohort study of birthing parent–child pairs from 8 cohorts across the US, we found that children born to birthing parents consuming a high-quality prenatal diet (as assessed via the 2015 HEI) were less likely to be born LGA, experience rapid infant growth between birth and ages 6 and 24 months, or experience slow growth between birth and ages 6, 12, and 24 months. However, associations of inflammatory diet, as assessed by the EDIP, with birth size and infant growth were inconsistent. Children born to birthing parents with a less inflammatory prenatal dietary pattern had increased odds of LGA and decreased odds of rapid growth between birth and age 6 months; however, this dietary pattern was also associated with 50% increased odds of slow growth from birth to age 24 months. These findings support a prenatal diet that aligns with US Dietary Guidelines (as measured by the HEI) to promote healthy birth weight and infant growth through age 24 months. More studies are needed to understand the potential benefits or harms of low-inflammatory prenatal dietary patterns in fetal and infant growth.^37^

Our findings are generally consistent with prior studies that examined similar dietary indices and infant size for gestational age at birth. Consistent with our findings, 3 studies in the US found inverse associations of healthier prenatal HEI-2010 index with infant birth weight, adiposity, and LGA.^38,39,40^ Consistent with prior studies, we found significant associations between AHEI-P and size for gestational age.^41,42^ Higher maternal dietary inflammatory index has been associated with an increased risk of SGA,^21^ we found that a less inflammatory dietary pattern according to the EDIP was associated with increased odds of LGA, suggesting that an inflammatory dietary pattern may inhibit infant growth and a less inflammatory pattern may promote growth.

Fewer studies have investigated the longer-term associations between prenatal diet quality and longer-term infant or child growth. One large study by Hu et al^43^ examined diet assessed via FFQ in the second trimester and data-derived dietary patterns and found pregnant participants with greater adherence to a fast-food pattern were more likely to have children in the increasing or high BMI trajectory from birth to age 4 years (OR, 1.32 [95% CI, 1.07-1.62]). This finding is generally consistent with our finding that a more healthful dietary pattern is associated with a reduced risk of rapid infant growth at ages 6 and 24 months. However, the study by Hu et al^43^ did not use an a priori–defined dietary pattern but rather derived it based on the available dietary data, making it not reproducible by other studies. We found that HEI during pregnancy was associated with decreased risk of extreme rapid infant growth from birth to 6 months and 24 months, which is associated with later-life obesity.^27^

The biologic mechanisms underlying the association of maternal diet with longer term infant growth remains to be clarified. However, animal studies have shown that prenatal overnutrition may be involved in the regulation of appetitive structures, mainly the hypothalamic neural network,^44,45^ as well as epigenetic changes in leptin gene expression in offspring, which may influence the offspring’s appetite and the development of metabolic diseases.^46^ Thus, infant appetite is potentially a mediator on the causal pathway from prenatal diet to infant growth. Future research is needed to quantify the mediating effects of infant appetite and infant feeding patterns.

We are unaware of other studies examining prenatal exposure to EDIP and infant growth. Project Viva found that children of pregnant individuals in the highest quartile of the dietary inflammatory index had higher BMI *z* score growth rates between ages 3 and 10 years compared with children of individuals in lowest quartile of the dietary inflammatory index.^47^ While we did not examine longer-term child growth beyond the first 2 years of life in this analysis, such associations will be important for ECHO to explore in future studies.^29^ It is also worth noting that the EDIP is a distinct measure of dietary inflammation from the dietary inflammatory index, and it was empirically derived based on its association with blood levels of the inflammatory biomarker, C-reactive protein (CRP).^19^ EDIP scores are cross-sectionally associated with activation of proinflammatory signaling^48^; higher leptin and lower adiponectin levels, indicating metabolic inflammation^49^; an unfavorable lipid profile^50,51^; and high-sensitivity CRP in pregnancy.^52^ We found that low EDIP levels were associated with increased risk of LGA and slow growth at age 24 months. Inflammatory markers, such as CRP, and inflammatory diets have been inversely associated with birth weight,^53^ which is hypothesized to be due to greater resistance to blood flow, consequently restricting fetal growth.^54^ It is possible that less inflammatory diets decrease inflammation and allow for more growth, but this warrants future study. The association with subsequent slow growth could be “catch down” growth, or slower rate of growth in infancy, common in LGA infants.^55^ However, these associations appear to be complex, as no clear trend was observed, and they warrant future study.

We found different associations of the EDIP and the HEI with outcomes at birth and 12 and 24 months. However, EDIP and HEI only share a few common dietary components,^19^ and there are many differences. For example, snacks, fruit juice, and pizza are considered anti-inflammatory in the EDIP, but would contribute to a lower HEI score.

Our study has several strengths. The prospective study design, including diverse cohorts from across the US, increases the generalizability of our study findings. We examined 2 dietary indices that are known to be associated with obesity and cardiovascular disease risk^43,51,56,57^ and that were defined a priori, making them generalizable when studying other populations. We assessed several potential confounders to assess the robustness of our study findings.

### Limitations

This study had several limitations. Dietary data were harmonized from different dietary assessment tools, including different types of food frequency questionnaires and 24-hour dietary recalls. This may have led to misclassification of our exposure and may have biased our results toward the null. The EDIP is not validated for use with 24-hour recall data; therefore, EDIP could not be calculated on a subset of our cohort. We lacked information on infant feeding, which may have moderated the effects of prenatal dietary exposures and should be examined in future studies.

## Conclusions

In this cohort study among a large and diverse population of pregnant individuals from across the US, we found that consuming a healthier diet in pregnancy in adherence with current USDA guidelines was associated with having an infant with reduced risk of LGA and with greater odds of having a healthier growth trajectory during the first 2 years of life. Our findings support the recommendation of a healthy diet based on the current guidelines (as measured by the HEI) during pregnancy, since it may reduce patterns of infant growth outside reference ranges, which are risk factors associated with obesity. The associations between the EDIP and infant growth are less clear and warrant further study.

## Notes

Supplement 1. **eTable 1.** Comparison of Analytic Sample and Mother-Child Pairs Excluded from Analysis **eTable 2.** Description of Dietary Assessments by Cohort **eTable 3.** Association Between Prenatal Alternative Healthy Eating Index Pregnancy (AHEI-P) Dietary Pattern and Size for Gestational Age at Birth Category **eTable 4.** Association Between Prenatal Empirical Dietary Inflammatory Pattern (EDIP) and Size for Gestational Age at birth Category and Infant Growth at 6, 12 and 24 Months, by Quartile Of EDIP **eFigure 1.** Flow Diagram of Analytic Sample **eFigure 2.** Adjusted Odds Ratios of the Alternative Healthy Eating Index in Pregnancy and Infant Growth at 6, 12 and 24 Months **eFigure 3.** Adjusted Odds Ratios of the Alternative Healthy Eating Index in Pregnancy and Extremes of Infant Growth at 6, 12 and 24 Months

Supplement 2.
ECHO Cohort Consortium Members

Supplement 3.
Data Sharing Statement

## References

[1] Ogden CL, Carroll MD, Kit BK, Flegal KM. Prevalence of childhood and adult obesity in the United States, 2011-2012. JAMA. 2014;311(8):806-814. doi:10.1001/jama.2014.732 24570244 PMC4770258

[2] Schwarzenberg SJ, Georgieff MK; Committee on Nutrition. Advocacy for improving nutrition in the first 1000 days to support childhood development and adult health. Pediatrics. 2018;141(2):e20173716. doi:10.1542/peds.2017-3716 29358479

[3] Parlee SD, MacDougald OA. Maternal nutrition and risk of obesity in offspring: the Trojan horse of developmental plasticity. Biochim Biophys Acta. 2014;1842(3):495-506. doi:10.1016/j.bbadis.2013.07.007 23871838 PMC3855628

[4] Symonds ME, Stephenson T, Gardner DS, Budge H. Long-term effects of nutritional programming of the embryo and fetus: mechanisms and critical windows. Reprod Fertil Dev. 2007;19(1):53-63. doi:10.1071/RD06130 17389135

[5] Stephenson J, Heslehurst N, Hall J, . Before the beginning: nutrition and lifestyle in the preconception period and its importance for future health. Lancet. 2018;391(10132):1830-1841. doi:10.1016/S0140-6736(18)30311-8 29673873 PMC6075697

[6] Dabelea D, Harrod CS. Role of developmental overnutrition in pediatric obesity and type 2 diabetes. Nutr Rev. 2013;71(suppl 1):S62-S67. doi:10.1111/nure.12061 24147926

[7] Oken E, Kleinman KP, Olsen SF, Rich-Edwards JW, Gillman MW. Associations of seafood and elongated n-3 fatty acid intake with fetal growth and length of gestation: results from a US pregnancy cohort. Am J Epidemiol. 2004;160(8):774-783. doi:10.1093/aje/kwh282 15466500 PMC1994920

[8] Zhu Y, Olsen SF, Mendola P, . Maternal dietary intakes of refined grains during pregnancy and growth through the first 7 y of life among children born to women with gestational diabetes. Am J Clin Nutr. 2017;106(1):96-104. doi:10.3945/ajcn.116.136291 28592607 PMC5486192

[9] Zhu Y, Olsen SF, Mendola P, . Maternal consumption of artificially sweetened beverages during pregnancy, and offspring growth through 7 years of age: a prospective cohort study. Int J Epidemiol. 2017;46(5):1499-1508. doi:10.1093/ije/dyx095 28586472 PMC5837735

[10] Zhu Y, Olsen SF, Mendola P, . Growth and obesity through the first 7 y of life in association with levels of maternal glycemia during pregnancy: a prospective cohort study. Am J Clin Nutr. 2016;103(3):794-800. doi:10.3945/ajcn.115.121780 26817507 PMC4763496

[11] Zheng M, Lamb KE, Grimes C, . Rapid weight gain during infancy and subsequent adiposity: a systematic review and meta-analysis of evidence. Obes Rev. 2018;19(3):321-332. doi:10.1111/obr.12632 29052309 PMC6203317

[12] Arisaka O, Ichikawa G, Koyama S, Sairenchi T. Childhood obesity: rapid weight gain in early childhood and subsequent cardiometabolic risk. Clin Pediatr Endocrinol. 2020;29(4):135-142. doi:10.1297/cpe.29.135 33088012 PMC7534524

[13] Cauzzo C, Chiavaroli V, Di Valerio S, Chiarelli F. Birth size, growth trajectory and later cardio-metabolic risk. Front Endocrinol (Lausanne). 2023;14:1187261. doi:10.3389/fendo.2023.1187261 37342257 PMC10277632

[14] Woo Baidal JA, Locks LM, Cheng ER, Blake-Lamb TL, Perkins ME, Taveras EM. Risk factors for childhood obesity in the first 1,000 days: a systematic review. Am J Prev Med. 2016;50(6):761-779. doi:10.1016/j.amepre.2015.11.012 26916261

[15] Olsen SF, Grandjean P, Weihe P, Viderø T. Frequency of seafood intake in pregnancy as a determinant of birth weight: evidence for a dose dependent relationship. J Epidemiol Community Health. 1993;47(6):436-440. doi:10.1136/jech.47.6.436 8120495 PMC1059854

[16] Mitchell EA, Robinson E, Clark PM, . Maternal nutritional risk factors for small for gestational age babies in a developed country: a case-control study. Arch Dis Child Fetal Neonatal Ed. 2004;89(5):F431-F435. doi:10.1136/adc.2003.036970 15321964 PMC1721755

[17] van Eijsden M, Hornstra G, van der Wal MF, Vrijkotte TG, Bonsel GJ. Maternal n-3, n-6, and trans fatty acid profile early in pregnancy and term birth weight: a prospective cohort study. Am J Clin Nutr. 2008;87(4):887-895. doi:10.1093/ajcn/87.4.887 18400711

[18] Krebs-Smith SM, Pannucci TE, Subar AF, . Update of the Healthy Eating Index: HEI-2015. J Acad Nutr Diet. 2018;118(9):1591-1602. doi:10.1016/j.jand.2018.05.021 30146071 PMC6719291

[19] Tabung FK, Smith-Warner SA, Chavarro JE, . Development and validation of an empirical Dietary Inflammatory Index. J Nutr. 2016;146(8):1560-1570. doi:10.3945/jn.115.228718 27358416 PMC4958288

[20] Lee DH, Li J, Li Y, . Dietary inflammatory and insulinemic potential and risk of type 2 diabetes: results from three prospective U.S. cohort studies. Diabetes Care. 2020;43(11):2675-2683. doi:10.2337/dc20-0815 32873589 PMC7576428

[21] Souza MDDC, Ferreira LB, Dos Santos LC. Dietary Inflammatory Index during pregnancy is associated with birth weight and child anthropometry up to 10 years old: a systematic review and meta-analysis. Nutr Res. 2023;114:81-97. doi:10.1016/j.nutres.2023.04.009 37209507

[22] Gillman MW, Blaisdell CJ. Environmental influences on child health outcomes, a research program of the National Institutes of Health. Curr Opin Pediatr. 2018;30(2):260-262. doi:10.1097/MOP.0000000000000600 29356702 PMC6020137

[23] Knapp EAKA, Kress AM, Parker CB, . The Environmental Influences on Child Health Outcomes (ECHO)–wide cohort. Am J Epidemiol. 2023;192(8):1249-1263. doi:10.1093/aje/kwad071 36963379 PMC10403303

[24] Group WHOMGRS; WHO Multicentre Growth Reference Study Group. WHO child growth standards based on length/height, weight and age. Acta Paediatr Suppl. 2006;450:76-85. 16817681 10.1111/j.1651-2227.2006.tb02378.x

[25] Centers for Disease Control and Prevention. A SAS Program for the WHO Growth Charts (ages 0 to <2 years). Centers for Disease Control and Prevention. Updated January 13, 2022. Accessed October 22, 2024. https://www.cdc.gov/growth-chart-training/hcp/computer-programs/sas-who.html?CDC_AAref_Val=https://www.cdc.gov/nccdphp/dnpao/growthcharts/resources/sas-who.htm

[26] Papageorghiou AT, Kennedy SH, Salomon LJ, ; International Fetal and Newborn Growth Consortium for the 21(st) Century (INTERGROWTH-21[st]). The INTERGROWTH-21st fetal growth standards: toward the global integration of pregnancy and pediatric care. Am J Obstet Gynecol. 2018;218(2S):S630-S640. doi:10.1016/j.ajog.2018.01.011 29422205

[27] Wang G, Johnson S, Gong Y, . Weight gain in infancy and overweight or obesity in childhood across the gestational spectrum: a prospective birth cohort study. Sci Rep. 2016;6:29867. doi:10.1038/srep29867 27417566 PMC4945912

[28] Jacobson LP, Lau B, Catellier D, Parker CB. An Environmental Influences on Child Health Outcomes viewpoint of data analysis centers for collaborative study designs. Curr Opin Pediatr. 2018;30(2):269-275. doi:10.1097/MOP.0000000000000602 29474274 PMC5877813

[29] Bragg MG, Westlake M, Alshawabkeh AN, ; program collaborators for Environmental influences on Child Health Outcomes. Opportunities for examining child health impacts of early-life nutrition in the ECHO Program: maternal and child dietary intake data from pregnancy to adolescence. Curr Dev Nutr. 2023;7(11):102019. doi:10.1016/j.cdnut.2023.102019 38035205 PMC10681943

[30] Guenther PM, Kirkpatrick SI, Reedy J, . The Healthy Eating Index-2010 is a valid and reliable measure of diet quality according to the 2010 Dietary Guidelines for Americans. J Nutr. 2014;144(3):399-407. doi:10.3945/jn.113.183079 24453128 PMC3927552

[31] Guenther PM, Casavale KO, Reedy J, . Update of the Healthy Eating Index: HEI-2010. J Acad Nutr Diet. 2013;113(4):569-580. doi:10.1016/j.jand.2012.12.016 23415502 PMC3810369

[32] US Department of Agriculture. The Healthy Eating Index. Accessed October 17, 2024. https://www.fns.usda.gov/cnpp/healthy-eating-index-hei

[33] Rifas-Shiman SL, Rich-Edwards JW, Kleinman KP, Oken E, Gillman MW. Dietary quality during pregnancy varies by maternal characteristics in Project Viva: a US cohort. J Am Diet Assoc. 2009;109(6):1004-1011. doi:10.1016/j.jada.2009.03.001 19465182 PMC4098830

[34] Flanagin A, Frey T, Christiansen SL; AMA Manual of Style Committee. Updated Guidance on the Reporting of Race and Ethnicity in Medical and Science Journals. JAMA. 2021;326(7):621-627. doi:10.1001/jama.2021.13304 34402850

[35] Avalos LA, Caan B, Nance N, . Prenatal depression and diet quality during pregnancy. J Acad Nutr Diet. 2020;120(6):972-984. doi:10.1016/j.jand.2019.12.011 32063456 PMC8006531

[36] Oshiro CE, Novotny R, Grove JS, Hurwitz EL. Race/ethnic differences in birth size, infant growth, and body mass index at age five years in children in Hawaii. Child Obes. 2015;11(6):683-690. doi:10.1089/chi.2015.0027 26561722

[37] Ong KK. Healthy growth and development. Nestle Nutr Inst Workshop Ser. 2017;87:141-151. doi:10.1159/000448964 28315895

[38] Shapiro AL, Kaar JL, Crume TL, . Maternal diet quality in pregnancy and neonatal adiposity: the Healthy Start Study. Int J Obes (Lond). 2016;40(7):1056-1062. doi:10.1038/ijo.2016.79 27133623 PMC5356926

[39] Grandy M, Snowden JM, Boone-Heinonen J, Purnell JQ, Thornburg KL, Marshall NE. Poorer maternal diet quality and increased birth weight. J Matern Fetal Neonatal Med. 2018;31(12):1613-1619. doi:10.1080/14767058.2017.1322949 28514885 PMC5694379

[40] Zhu Y, Hedderson MM, Sridhar S, Xu F, Feng J, Ferrara A. Poor diet quality in pregnancy is associated with increased risk of excess fetal growth: a prospective multi-racial/ethnic cohort study. Int J Epidemiol. 2019;48(2):423-432. doi:10.1093/ije/dyy285 30590563 PMC6469312

[41] Rifas-Shiman SL, Rich-Edwards JW, Kleinman KP, Oken E, Gillman MW. Dietary quality during pregnancy varies by maternal characteristics in Project Viva: a US cohort. J Am Diet Assoc. 2009;109(6):1004-1011. doi:10.1016/j.jada.2009.03.001 19465182 PMC4098830

[42] Poon AK, Yeung E, Boghossian N, Albert PS, Zhang C. Maternal Dietary patterns during third trimester in association with birthweight characteristics and early infant growth. Scientifica (Cairo). 2013;2013:786409. doi:10.1155/2013/786409 24490111 PMC3893866

[43] Hu Z, Tylavsky FA, Kocak M, . Effects of maternal dietary patterns during pregnancy on early childhood growth trajectories and obesity risk: the CANDLE Study. Nutrients. 2020;12(2):465. doi:10.3390/nu12020465 32069778 PMC7071328

[44] McMillen IC, Adam CL, Mühlhäusler BS. Early origins of obesity: programming the appetite regulatory system. J Physiol. 2005;565(Pt 1):9-17. doi:10.1113/jphysiol.2004.081992 15705647 PMC1464497

[45] Plagemann A, Harder T, Rake A, . Perinatal elevation of hypothalamic insulin, acquired malformation of hypothalamic galaninergic neurons, and syndrome X-like alterations in adulthood of neonatally overfed rats. Brain Res. 1999;836(1-2):146-155. doi:10.1016/S0006-8993(99)01662-5 10415413

[46] Masuyama H, Mitsui T, Nobumoto E, Hiramatsu Y. The effects of high-fat diet exposure in utero on the obesogenic and diabetogenic traits through epigenetic changes in adiponectin and leptin gene expression for multiple generations in female mice. Endocrinology. 2015;156(7):2482-2491. doi:10.1210/en.2014-2020 25853666

[47] Monthé-Drèze C, Rifas-Shiman SL, Aris IM, . Maternal diet in pregnancy is associated with differences in child body mass index trajectories from birth to adolescence. Am J Clin Nutr. 2021;113(4):895-904. doi:10.1093/ajcn/nqaa398 33721014 PMC8023853

[48] Locksley RM, Killeen N, Lenardo MJ. The TNF and TNF receptor superfamilies: integrating mammalian biology. Cell. 2001;104(4):487-501. doi:10.1016/S0092-8674(01)00237-9 11239407

[49] Fantuzzi G. Adipose tissue, adipokines, and inflammation. J Allergy Clin Immunol. 2005;115(5):911-919. doi:10.1016/j.jaci.2005.02.023 15867843

[50] Anand SS, Hawkes C, de Souza RJ, . Food consumption and its impact on cardiovascular disease: importance of solutions focused on the globalized food system: a report from the workshop convened by the World Heart Federation. J Am Coll Cardiol. 2015;66(14):1590-1614. doi:10.1016/j.jacc.2015.07.050 26429085 PMC4597475

[51] Li J, Lee DH, Hu J, . Dietary inflammatory potential and risk of cardiovascular disease among men and women in the U.S. J Am Coll Cardiol. 2020;76(19):2181-2193. doi:10.1016/j.jacc.2020.09.535 33153576 PMC7745775

[52] Yin WJ, Yu LJ, Wu L, . Adequate 25(OH)D moderates the relationship between dietary inflammatory potential and cardiovascular health risk during the second trimester of pregnancy. Front Nutr. 2022;9:952652. doi:10.3389/fnut.2022.952652 35967812 PMC9372498

[53] Yang Y, Kan H, Yu X, Yang Y, Li L, Zhao M. Relationship between dietary inflammatory index, hs-CRP level in the second trimester and neonatal birth weight: a cohort study. J Clin Biochem Nutr. 2020;66(2):163-167. doi:10.3164/jcbn.19-100 32231414 PMC7093294

[54] Xu F, Ren ZX, Zhong XM, Zhang Q, Zhang JY, Yang J. Intrauterine inflammation damages placental angiogenesis via Wnt5a-Flt1 activation. Inflammation. 2019;42(3):818-825. doi:10.1007/s10753-018-0936-y 30543046

[55] Taal HR, Vd Heijden AJ, Steegers EA, Hofman A, Jaddoe VW. Small and large size for gestational age at birth, infant growth, and childhood overweight. Obesity (Silver Spring). 2013;21(6):1261-1268. doi:10.1002/oby.20116 23666877

[56] Zhang Y, Lu C, Li X, . Healthy Eating Index-2015 and predicted 10-year cardiovascular disease risk, as well as heart age. Front Nutr. 2022;9:888966. doi:10.3389/fnut.2022.888966 35903444 PMC9315384

[57] Shi N, Aroke D, Jin Q, . Proinflammatory and hyperinsulinemic dietary patterns are associated with specific profiles of biomarkers predictive of chronic inflammation, glucose-insulin dysregulation, and dyslipidemia in postmenopausal women. Front Nutr. 2021;8:690428. doi:10.3389/fnut.2021.690428 34616762 PMC8488136

