# Supplementary material for: Prenatal Diet and Infant Growth From Birth to Age 24 Months
Source: JAMA Netw Open. 2024 Nov 21;7(11):e2445771. doi: 10.1001/jamanetworkopen.2024.45771 (PMC11582932; doi:10.1001/jamanetworkopen.2024.45771)
Supplement: Supplement 1. — eTable 1. Comparison of Analytic Sample and Mother-Child Pairs Excluded from Analysis eTable 2. Description of Dietary Assessments by Cohort eTable 3. Association Between Prenatal Alternative Healthy Eating Index Pregnancy (AHEI-P) Dietary Pattern and Size for Gestational Age at Birth Category eTable 4. Association Between Prenatal Empirical Dietary Inflammatory Pattern (EDIP) and Size for Gestational Age at birth Category and Infant Growth at 6, 12 and 24 Months, by Quartile Of EDIP eFigure 1. Flow Diagram of Analytic Sample eFigure 2. Adjusted Odds Ratios of the Alternative Healthy Eating Index in Pregnancy and Infant Growth at 6, 12 and 24 Months eFigure 3. Adjusted Odds Ratios of the Alternative Healthy Eating Index in Pregnancy and Extremes of Infant Growth at 6, 12 and 24 Months [file jamanetwopen-e2445771-s001.pdf]

Supplemental Online Content

Hedderson MM, Schuh HB, Knapp EA, et al; the ECHO Cohort Consortium. Prenatal diet and infant growth from birth to age 24 months. *JAMA Netw Open*. 2024;7(11):e2445771. doi:10.1001/jamanetworkopen.2024.45771

eTable 1. Comparison of Analytic Sample and Mother-Child Pairs Excluded from Analysis
eTable 2. Description of Dietary Assessments by Cohort
eTable 3. Association Between Prenatal Alternative Healthy Eating Index Pregnancy (AHEI-P) Dietary Pattern and Size for Gestational Age at Birth Category
eTable 4. Association Between Prenatal Empirical Dietary Inflammatory Pattern (EDIP) and Size for Gestational Age at birth Category and Infant Growth at 6, 12 and 24 Months, by Quartile Of EDIP
eFigure 1. Flow Diagram of Analytic Sample
eFigure 2. Adjusted Odds Ratios of the Alternative Healthy Eating Index in Pregnancy and Infant Growth at 6, 12 and 24 Months
eFigure 3. Adjusted Odds Ratios of the Alternative Healthy Eating Index in Pregnancy and Extremes of Infant Growth at 6, 12 and 24 Months

This supplemental material has been provided by the authors to give readers additional information about their work.

Supplementary eTable 1. Comparison of Analytic Sample and Mother-Child Pairs Excluded from Analysis

	**Total Included in Sample**	**Excluded**
**Birthing parent-Child Pairs, N**	2,854	7,707
**Child Characteristics**		
**Birth year**		
2007-2011	952 (33.4%)	2,898 (37.6%)
2012-2016	1,275 (44.7%)	3,754 (48.7%)
2017-2021	627 (22.0%)	1,055 (13.7%)
**Child sex (at birth), female**	1,390 (48.7%)	3,712 (48.2%)
**Child race/ethnicity**		
White	664 (23.3%)	4,377 (56.8%)
Black	640 (22.4%)	822 (10.7%)
Asian, Native Hawaiian or Pacific Islander	225 (7.9%)	281 (3.6%)
Hispanic	1,022 (35.8%)	1,185 (15.4%)
Other or multiracial	224 (7.8%)	983 (12.8%)
Missing	79 (2.8%)	59 (0.8%)
**Birthweight (grams)**	3325 (3040-3650)	3430 (3130-3742)
**Size at birth**		
Appropriate for gestational age	2,294 (80.4%)	5,812 (75.4%)
Small for gestational age	155 (5.4%)	298 (3.9%)
Large for gestational age	404 (14.2%)	1,509 (19.6%)
Missing	<5	88 (1.1%)
**Preterm birth**	138 (4.8%)	326 (4.2%)
**Maternal Characteristics**		
**Maternal age at delivery**		
<25	586 (20.5%)	1,370 (17.8%)
25-29	763 (26.7%)	2,113 (27.4%)
30-34	917 (32.1%)	2,531 (32.8%)
35+	588 (20.6%)	1,495 (19.4%)
**Pre-pregnancy BMI**		
Normal (18.5-24.9, kg/m2)	1,156 (40.5%)	3,092 (40.1%)
Underweight (<18.5, kg/m2)	74 (2.6%)	176 (2.3%)
Overweight (25-29.9, kg/m2)	767 (26.9%)	1,741 (22.6%)
Obese (30+, kg/m2)	844 (29.6%)	1,724 (22.4%)
Missing	13 (0.5%)	974 (12.6%)
**Maternal educational status, highest**		
<College degree	1,562 (54.7%)	1,212 (15.7%)
College degree	744 (26.1%)	1,029 (13.4%)
Graduate degree	501 (17.6%)	866 (11.2%)
Missing	47 (1.6%)	4,600 (59.7%)
**Prenatal tobacco/nicotine use**	135 (4.7%)	999 (13.0%)
**Missing**	<5	655 (8.5%)

Supplemental Table 2. Description of Dietary Assessments by Cohort

		1st Trimester			2nd Trimester			3rd Trimester		
Cohort	Diet Assessment	24-hr	FFQ	Total	24-hr	FFQ	Total	24-hr	FFQ	Total
1	ASA24[1] (2011)	48	0	48	237	0	237	5	0	5
2	ASA24[1] (2016)				<5	0	<5	13	0	13
3	Block-Bodnar Questionnaire for Women of Reproductive Age	0	17	17	0	12	12			
4	Block-Bodnar Questionnaire for Women of Reproductive Age	0	846	846	0	423	423	0	<5	<5
5	ASA24[1] (2016)<br>ASA24[1] (2018)				<5	0	<5	237	0	237
6	Block-Bodnar Questionnaire for Women of Reproductive Age				0	689	689	0	96	96
7	Block-Bodnar Questionnaire for Women of Reproductive Age				0	9	9	0	8	8
8	Block-Bodnar Questionnaire for Women of Reproductive Age	0	24	24	0	80	80	0	90	90
	Total	48	887	935	<255	1213	<1468	255	<200	<455

Supplemental **Table 3.** Association between prenatal Alternative Healthy Eating Index Pregnancy (AHEI-P)[c] dietary pattern and size for gestational age at birth category

	Adjusted Odd ration (95% CI)[a]
Size for Gestational Age	High Alternative Healthy Eating Index (AHEI)[c]
	n=2711
Appropriate	Reference
Small[a]	0.86 [0.71,1.04]
Large[b]	0.73 [0.49,1.09]

[a] Multinomial logistic regression models adjusted for maternal education, child sex, maternal age at delivery, pre-pregnancy Body Mass Index (BMI), child race and ethnicity, prenatal tobacco use, dietary assessment (food frequency questionnaire or 24-hour recall), and trimester of dietary assessment. Robust standard errors clustered on cohort were included in the estimate of the to account for correlation of children within the same cohort.
[b] High AHEI-P was defined as a score >76.3% using the mean of the highest quartile cut-off
[c] using Intergrowth-21st reference less than and not including 10th percentile; b: using Intergrowth-21st reference greater than and not including 90th percentile.

Supplemental Table 4. Association between prenatal Empirical Dietary Inflammatory Pattern (EDIP)[a] and size for gestational age at birth category and infant growth at 6, 12 and 24 months, by quartile of EDIP.

	Birthweight		
Size for gestational age	n=2159		
**Small for gestational age[b]***			
Most Inflammatory (Q4)	REF		
Inflammatory (Q3)	0.90 [0.63, 1.30]		
Less Inflammatory (Q2)	1.06 [0.67, 1.69]		
Least Inflammatory (Q1)	0.83 [0.62, 1.12]		
**Large for gestational age[c]***			
Most Inflammatory (Q4)	REF		
Inflammatory (Q3)	0.89 [0.51, 1.55]		
Less Inflammatory (Q2)	0.73 [0.46, 1.17]		
Least Inflammatory (Q1)	0.90 [0.71, 1.15]		
	6 months	12 months	24 months
	n=1303	n=1602	n=1115
**Slow growth****			
Most Inflammatory (Q4)	REF	REF	REF
Inflammatory (Q3)	0.88[0.69, 1.11]	1.11 [0.94, 1.30]	0.91 [0.72, 1.16]
Less Inflammatory (Q2)	0.77 [0.57, 1.03]	1.31 [1.01, 1.69]	1.23 [0.93, 1.63]
Least Inflammatory (Q1)	0.74 [0.64, 0.86]	1.38 [1.12, 1.69]	1.14 [0.91, 1.44]

**Rapid growth****			
Most Inflammatory (Q4)	REF	REF	REF
Inflammatory (Q3)	0.92 [0.67, 1.27]	0.88 [0.59, 1.33]	1.01 [0.58, 1.76]
Less Inflammatory (Q2)	0.87 [0.81, 0.95]	0.95 [0.83, 1.10]	1.01 [0.93, 1.10]
Least Inflammatory (Q1)	0.91 [0.78, 1.07]	1.00 [0.73, 1.38]	1.12 [0.85, 1.48]

[a] EDIP quartile, mean(SD): Most Inflammatory: 64.7(0.6); Inflammatory 64.0(0.1); Less Inflammatory: 63.8(0.1); Least Inflammatory: 63.5(0.3)

*Reference is Appropriate for Gestational Age [b]: using Intergrowth-21st reference less than and not including 10th percentile; [c:] using Intergrowth-21st reference greater than and not including 90th percentile. Multinomial logistic regression models adjusted for maternal education, child sex, maternal age at delivery, pre-pregnancy Body Mass Index (BMI), child race and ethnicity, prenatal tobacco use, dietary assessment (food frequency questionnaire or 24-hour recall), and trimester of dietary assessment. Robust standard errors clustered on cohort were included in the estimate of the to account for correlation of children within the same cohort.

**Reference is Appropriate growth defined as a difference in weight-for-length z-score between birth and 6, 12, or 24 months of -0.67 to 0.67.

Supplemental Figure 1.

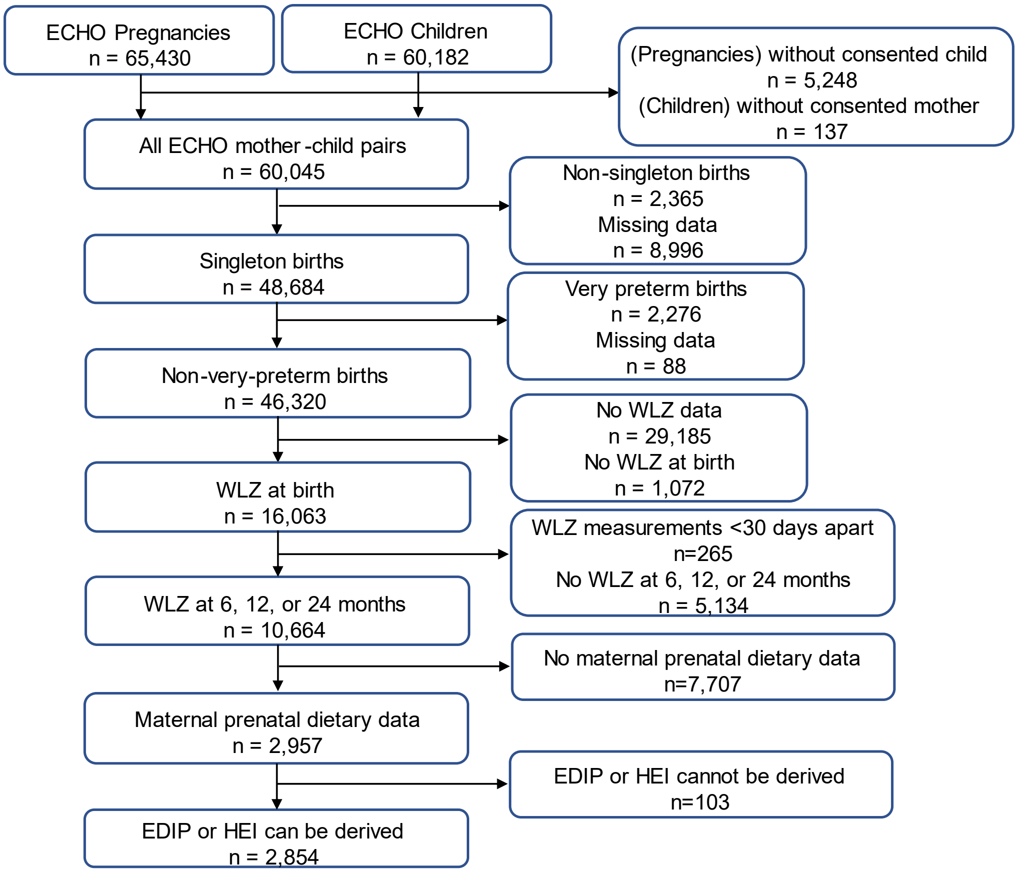

Supplemental Figure 2.

Model	aOR (95% CI)
Slow Growth 6 Months	1.50 (0.72–3.13)
Slow Growth 12 Months	1.22 (1.17–1.26)
Slow Growth 24 Months	0.58 (0.47–0.71)
Appropriate Growth 6 Months	Reference
Appropriate Growth 12 Months	Reference
Appropriate Growth 24 Months	Reference
Rapid Growth 6 Months	1.06 (0.75–1.49)
Rapid Growth 12 Months	0.82 (0.54–1.26)
Rapid Growth 24 Months	0.90 (0.71–1.14)

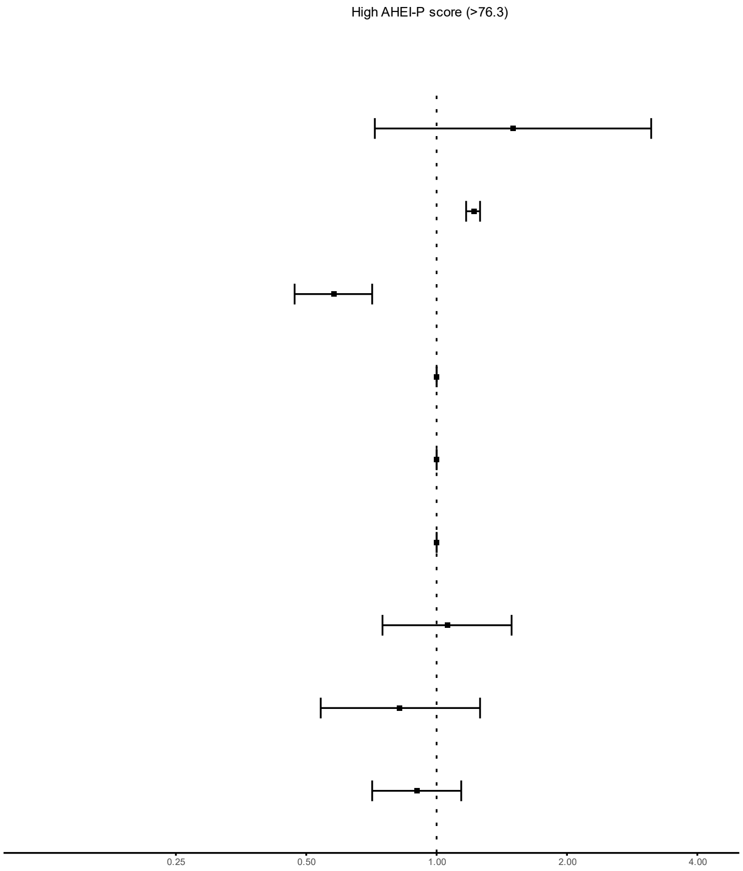

© 2024 Hedderson MM et al. *JAMA Network Open.*

Supplemental Figure 3.

Model	aOR (95%CI)
Extremely Slow Growth 6 Months	1.59 (0.94–2.70)
Extremely Slow Growth 12 Months	0.84 (0.48–1.46)
Extremely Slow Growth 24 Months	0.58 (0.33–0.99)
Slow Growth 6 Months	1.40 (0.51–3.83)
Slow Growth 12 Months	1.49 (1.14–1.94)
Slow Growth 24 Months	0.59 (0.35–1.01)
Appropriate Growth 6 Months	Reference
Appropriate Growth 12 Months	Reference
Appropriate Growth 24 Months	Reference
Rapid Growth 6 Months	1.16 (1.03–1.31)
Rapid Growth 12 Months	0.94 (0.89–0.98)
Rapid Growth 24 Months	1.00 (0.63–1.58)
Extremely Rapid Growth 6 Months	1.00 (0.62–1.60)
Extremely Rapid Growth 12 Months	0.78 (0.43–1.41)
Extremely Rapid Growth 24 Months	0.86 (0.73–1.00)

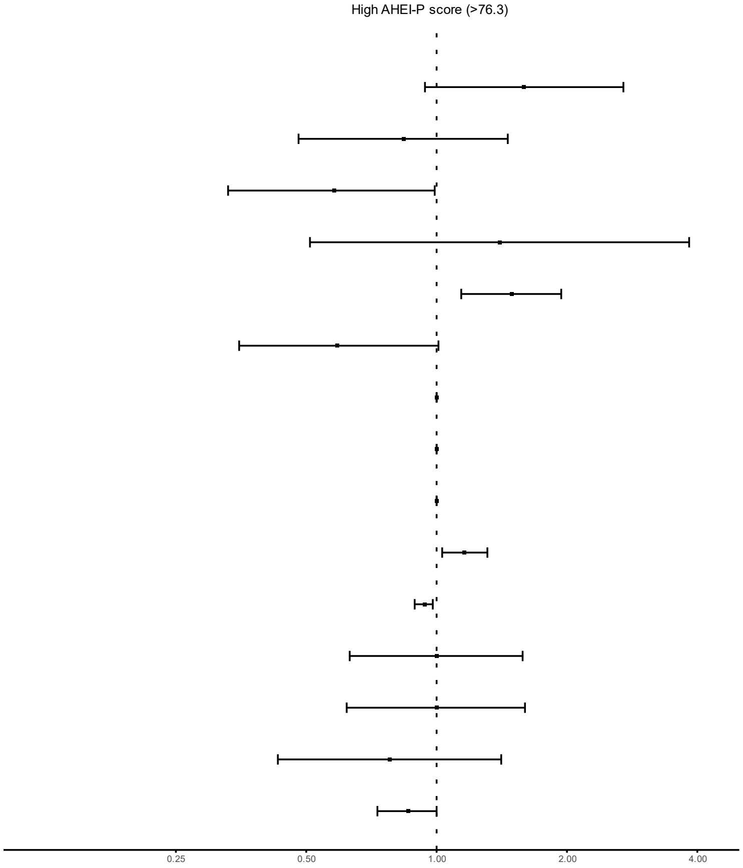

© 2024 Hedderson MM et al. *JAMA Network Open.*
