# Supplementary material for: Prenatal Diet and Infant Growth From Birth to Age 24 Months
Source: JAMA Netw Open. 2024 Nov 21;7(11):e2445771. doi: 10.1001/jamanetworkopen.2024.45771 (PMC11582932; doi:10.1001/jamanetworkopen.2024.45771)
Supplement: Supplement 2. — ECHO Cohort Consortium Members [file jamanetwopen-e2445771-s002.pdf]

Supplemental Online Content: Nonauthor Collaborators
*First name, last name, and suffix (if applicable) are required and will appear in PubMed.

*Group Name(s): ECHO Cohort Consortium							
***First Name and Middle Initial(s)**	***Last Name**	***Suffix (eg, Jr, III)**	Academic Degrees	Institution	Location (city, state/province, country)	Role or Contribution, eg, chair, principal investigator	Group (if more than 1 Group listed in the byline) and/or Subgroup (eg, Steering Committee)
P Brian	Smith		MD, MPH, MHS	Duke Clinical Research Institute, Duke University School of Medicine	Durham, North Carolina, USA	ECHO Coordinating Center Principal Investigator	U2COD023375 (Coordinating Center)
L Kristin	Newby		MD, MHS	Duke Clinical Research Institute, Duke University School of Medicine	Durham, North Carolina, USA	ECHO Coordinating Center Principal Investigator	U2COD023375 (Coordinating Center)
Linda	Adair		PhD	Gillings School of Global Public Health, University of North Carolina at Chapel Hill	Chapel Hill, North Carolina, USA	ECHO Coordinating Center Principal Investigator	U2COD023375 (Coordinating Center)
Lisa P.	Jacobson		ScD	Johns Hopkins University, Bloomberg School of Public Health	Baltimore, Maryland, USA	ECHO Data Analysis Center Principal Investigator	U24OD023382 (Data Analysis Center)
Diane	Catellier		DrPH	Research Triangle Institute	Research Triangle Park, North Carolina, USA	ECHO Data Analysis Center Principal Investigator	U24OD023382 (Data Analysis Center)
Monica	McGrath		ScD	Johns Hopkins University, Bloomberg School of Public Health	Baltimore, Maryland, USA	ECHO Johns Hopkins University Data Analysis Center Director Co-Investigator	U24OD023382 (Data Analysis Center)
Christian	Douglas		DrPH	Research Triangle Institute	Research Triangle Park, North Carolina, USA	ECHO RTI Data Analysis Center Director Co-Investigator	U24OD023382 (Data Analysis Center)
Priya	Duggal		PhD	Johns Hopkins University, Bloomberg School of Public Health	Baltimore, Maryland, USA	ECHO Data Analysis Center Genetics Methods Lead, Co-Investigator	U24OD023382 (Data Analysis Center)

Supplemental Online Content: Nonauthor Collaborators
*First name, last name, and suffix (if applicable) are required and will appear in PubMed.

***First Name and Middle Initial(s)**	***Last Name**	***Suffix (eg, Jr, III)**	Academic Degrees	Institution	Location (city, state/province, country)	Role or Contribution, eg, chair, principal investigator	Group (if more than 1 Group listed in the byline) and/or Subgroup (eg, Steering Committee)
Emily	Knapp		PhD	Johns Hopkins University, Bloomberg School of Public Health	Baltimore, Maryland, USA	ECHO Data Analysis Center Co-Investigator	U24OD023382 (Data Analysis Center)
Amii	Kress		PhD	Johns Hopkins University, Bloomberg School of Public Health	Baltimore, Maryland, USA	ECHO Data Analysis Center General Methods Co-Investigator	U24OD023382 (Data Analysis Center)
Courtney K.	Blackwell		PhD	Feinberg School of Medicine, Northwestern University	Chicago, Illinois, USA	Measurement Core Co-Investigator	U24OD023319 with co-funding from the Office of Behavioral and Social Science Research (Measurement Core)
Maxwell A.	Mansolf		PhD	Feinberg School of Medicine, Northwestern University	Chicago, Illinois, USA	Measurement Core Co-Investigator	U24OD023319 with co-funding from the Office of Behavioral and Social Science Research (Measurement Core)
Jin-Shei	Lai		PhD	Feinberg School of Medicine, Northwestern University	Chicago, Illinois, USA	Measurement Core Co-Investigator	U24OD023319 with co-funding from the Office of Behavioral and Social Science Research (Measurement Core)
Emily	Ho		PhD	Feinberg School of Medicine, Northwestern University	Chicago, Illinois, USA	Measurement Core Co-Investigator	U24OD023319 with co-funding from the Office of Behavioral and Social Science Research (Measurement Core)

Supplemental Online Content: Nonauthor Collaborators
*First name, last name, and suffix (if applicable) are required and will appear in PubMed.

***First Name and Middle Initial(s)**	***Last Name**	***Suffix (eg, Jr, III)**	Academic Degrees	Institution	Location (city, state/province, country)	Role or Contribution, eg, chair, principal investigator	Group (if more than 1 Group listed in the byline) and/or Subgroup (eg, Steering Committee)
David	Cella		PhD	Feinberg School of Medicine, Northwestern University	Chicago, Illinois, USA	Measurement Core Principal Investigator	U24OD023319 with co-funding from the Office of Behavioral and Social Science Research (Measurement Core)
Richard	Gershon		PhD	Feinberg School of Medicine, Northwestern University	Chicago, Illinois, USA	Measurement Core Principal Investigator	U24OD023319 with co-funding from the Office of Behavioral and Social Science Research (Measurement Core)
Michelle L.	Macy		MD	Feinberg School of Medicine, Northwestern University and Ann & Robert H. Lurie Children's Hospital of Chicago	Chicago, Illinois, USA	Measurement Core Co-Investigator	U24OD023319 with co-funding from the Office of Behavioral and Social Science Research (Measurement Core)
Suman R.	Das		PhD	Vanderbilt University Medical Center	Nashville, Tennessee, USA	ECHO Laboratory Core Principal Investigator	U24OD035523 (Lab Core)
Jane E.	Freedman		MD	Vanderbilt University Medical Center	Nashville, Tennessee, USA	ECHO Laboratory Core Principal Investigator	U24OD035523 (Lab Core)
Simon A.	Mallal		MBBS	Vanderbilt University Medical Center	Nashville, Tennessee, USA	ECHO Laboratory Core Principal Investigator	U24OD035523 (Lab Core)
John A.	McLean		PhD	Vanderbilt University	Nashville, Tennessee, USA	ECHO Laboratory Core Principal Investigator	U24OD035523 (Lab Core)
Ravi V.	Shah		MD	Vanderbilt University Medical Center	Nashville, Tennessee, USA	ECHO Laboratory Core Principal Investigator	U24OD035523 (Lab Core)

Supplemental Online Content: Nonauthor Collaborators
*First name, last name, and suffix (if applicable) are required and will appear in PubMed.

***First Name and Middle Initial(s)**	***Last Name**	***Suffix (eg, Jr, III)**	Academic Degrees	Institution	Location (city, state/province, country)	Role or Contribution, eg, chair, principal investigator	Group (if more than 1 Group listed in the byline) and/or Subgroup (eg, Steering Committee)
Meghan H.	Shilts		MHS	Vanderbilt University Medical Center	Nashville, Tennessee, USA	ECHO Laboratory Core Principal Investigator Admin Designee	U24OD035523 (Lab Core)
Akram N.	Alshawabkeh		PhD	Northeastern University	Boston, Massachusetts, USA	ECHO Cohort Study Site Principal Investigator	UG3/UH3OD023251 (Akram Alshawabkeh)
Jose F.	Cordero		MD	University of Georgia	Athens, Georgia; USA	ECHO Cohort Study Site Co-Director	UG3/UH3OD023251 (Akram Alshawabkeh)
John	Meeker		ScD	University of Michigan	Ann Arbor, Michigan; USA	ECHO Cohort Study Site Co-Director	UG3/UH3OD023251 (Akram Alshawabkeh)
Leonardo	Trasande		MD, MPP	NYU Grossman School of Medicine	New York, New York, USA	ECHO Cohort Study Site Principal Investigator	UG3/UH3OD023305 (Leonardo Trasande)
Carlos A.	Camargo	Jr.	MD, DrPH	Massachusetts General Hospital, Harvard Medical School	Boston, Massachusetts, USA	ECHO Cohort Study Site Principal Investigator	UG3/UH3OD023253 (Carlos Camargo)
Kohei	Hasegawa		MD, PhD	Massachusetts General Hospital, Harvard Medical School	Boston, Massachusetts, USA	ECHO Cohort Study Site Co-Investigator	UG3/UH3OD023253 (Carlos Camargo)
Zhaozhong	Zhu		ScD	Massachusetts General Hospital, Harvard Medical School	Boston, Massachusetts, USA	ECHO Cohort Study Site Co-Investigator	UG3/UH3OD023253 (Carlos Camargo)
Ashley F.	Sullivan		MS, MPH	Massachusetts General Hospital, Harvard Medical School	Boston, Massachusetts, USA	ECHO Cohort Study Site Award Project Director	UG3/UH3OD023253 (Carlos Camargo)
Dana	Dabelea		MD, PhD	University of Colorado Anschutz Medical Campus	Aurora, Colorado, USA	ECHO Cohort Study Site Principal Investigator	UG3/UH3OD023248 and UG3OD035526 (Dana Dabelea)
Wei	Perng		PhD, MPH	University of Colorado Anschutz Medical Campus	Aurora, Colorado, USA	ECHO Cohort Study Site Principal Investigator	UG3/UH3OD023248 (Dana Dabelea)

Supplemental Online Content: Nonauthor Collaborators
*First name, last name, and suffix (if applicable) are required and will appear in PubMed.

***First Name and Middle Initial(s)**	***Last Name**	***Suffix (eg, Jr, III)**	Academic Degrees	Institution	Location (city, state/province, country)	Role or Contribution, eg, chair, principal investigator	Group (if more than 1 Group listed in the byline) and/or Subgroup (eg, Steering Committee)
Traci A.	Bekelman		PhD, MPH	University of Colorado Anschutz Medical Campus	Aurora, Colorado, USA	ECHO Cohort Study Site Principal Investigator	UG3/UH3OD023248 (Dana Dabelea)
Greta	Wilkening		PhD, MPH	University of Colorado Anschutz Medical Campus	Aurora, Colorado, USA	ECHO Cohort Study Site Co-Investigator	UG3/UH3OD023248 (Dana Dabelea)
Sheryl	Magzamen		PhD	Colorado School of Public Health, Colorado State University	Fort Collins, Colorado, USA	ECHO Cohort Study Site Co-Investigator	UG3OD035526 (Dana Dabelea)
Brianna F.	Moore		PhD, MS	University of Colorado Anschutz Medical Campus	Aurora, Colorado, USA	ECHO Cohort Study Site Principal Investigator	UG3OD035526 (Dana Dabelea)
Anne P.	Starling		PhD	University of North Carolina at Chapel Hill	Chapel Hill, North Carolina, USA	ECHO Cohort Study Site Principal Investigator	UG3OD035526 (Dana Dabelea)
Deborah J.	Rinehart		PhD	Denver Health and Hospital Authority	Denver, Colorado, USA	ECHO Cohort Study Site Co-Investigator	UG3OD035526 (Dana Dabelea)
Daphne	Koinis Mitchell		Ph.D	Rhode Island Hospital, The Alpert Medical School of Brown University	Providence, Rhode Island, USA	ECHO Cohort Study Site Principal Investigator	UG3/UH3OD023313 (Daphne Koinis Mitchell)
Viren	D'Sa		MD	Rhode Island Hospital, The Alpert Medical School of Brown University	Providence, Rhode Island, USA	ECHO Cohort Study Site Principal Investigator	UG3/UH3OD023313 (Daphne Koinis Mitchell)
Sean C.L.	Deoni		PhD	Bill & Melinda Gates Foundation	Seattle, Washington, USA	ECHO Cohort Study Site Principal Investigator	UG3/UH3OD023313 (Daphne Koinis Mitchell)
Hans-Georg	Mueller		PhD	University of California, Davis	Davis, California, USA	ECHO Cohort Study Site Co-Investigator	UG3/UH3OD023313 (Daphne Koinis Mitchell)
Cristiane S.	Duarte		PhD, MPH	Columbia University - NYSPI	New York, New York, USA	ECHO Cohort Study Site Principal Investigator	UH3OD023328 (Cristiane Duarte)

*First name, last name, and suffix (if applicable) are required and will appear in PubMed.

***First Name and Middle Initial(s)**	***Last Name**	***Suffix (eg, Jr, III)**	Academic Degrees	Institution	Location (city, state/province, country)	Role or Contribution, eg, chair, principal investigator	Group (if more than 1 Group listed in the byline) and/or Subgroup (eg, Steering Committee)
Catherine	Monk		PhD	Columbia University - NYSPI	New York, New York, USA	ECHO Cohort Study Site Principal Investigator	UH3OD023328 (Cristiane Duarte)
Glorisa	Canino		PhD	University of Puerto Rico, School of Medicine	Rio Piedras, Puerto Rico	ECHO Cohort Study Site Principal Investigator	UH3OD023328 (Cristiane Duarte)
Jonathan	Posner		MD	Duke University School of Medicine, Duke Psychiatry & Behavioral Sciences	Durham, North Carolina, USA	ECHO Cohort Study Site Principal Investigator	UH3OD023328 (Cristiane Duarte)
Tenneill	Murray		MPH	Columbia University - NYSPI	New York, New York, USA	ECHO Cohort Study Site Co-Director	UH3OD023328 (Cristiane Duarte)
Claudia	Lugo-Candelas		PhD	Columbia University - NYSPI	New York, New York, USA	ECHO Cohort Study Site Principal Investigator	UH3OD023328 (Cristiane Duarte)
Anne L.	Dunlop		MD, MPH	Emory University School of Medicine	Atlanta, Georgia, USA	ECHO Cohort Study Site Principal Investigator	UH3OD023318 (Anne Dunlop)
Patricia A.	Brennan		PhD	Emory University	Atlanta, Georgia, USA	ECHO Cohort Study Site Principal Investigator	UH3OD023318 (Anne Dunlop)
Christine	Hockett		PhD	Avera Research Institute; University of South Dakota School of Medicine	Rapid City, South Dakota, USA; Sioux Falls, South Dakota, USA	ECHO Cohort Study Site Principal Investigator	UG3/UH3OD023279 (Amy Elliott)
Amy	Elliott		PhD	Avera Research Institute ; University of South Dakota School of Medicine	Sioux Falls, South Dakota, USA	ECHO Cohort Study Site Principal Investigator	UG3/UH3OD023279 (Amy Elliott)
Assiamira	Ferrara		MD, PhD	Kaiser Permanente Northern California	Oakland, California, USA	ECHO Cohort Study Site Principal Investigator	UG3/UH3OD023289 (Assiamira Ferrara)

*First name, last name, and suffix (if applicable) are required and will appear in PubMed.

***First Name and Middle Initial(s)**	***Last Name**	***Suffix (eg, Jr, III)**	Academic Degrees	Institution	Location (city, state/province, country)	Role or Contribution, eg, chair, principal investigator	Group (if more than 1 Group listed in the byline) and/or Subgroup (eg, Steering Committee)
Lisa A.	Croen		PhD	Kaiser Permanente Northern California	Oakland, California, USA	ECHO Cohort Study Site Principal Investigator	UG3/UH3OD023342 (Kristen Lyall), UG3/UH3OD023290 (Julie Herbstman)
Monique M.	Hedderson		PhD	Kaiser Permanente Northern California	Oakland, California, USA	ECHO Cohort Study Site Principal Investigator	UG3/UH3OD023289 (Assiamira Ferrara), UG3OD035540 (Monique Marie Hedderson)
John	Ainsworth		PhD	University of Manchester	Manchester, United Kingdom	ECHO Cohort Study Site Principal Investigator	UG3/UH3OD023282 (James Gern)
Leonard B.	Bacharier		MD	Vanderbilt University Medical Center	Nashville, Tennessee, USA	ECHO Cohort Study Site Principal Investigator	UG3/UH3OD023282 (James Gern)
Casper G.	Bendixsen		PhD	Marshfield Clinic Research Institute	Marshfield, Wisconsin, USA	ECHO Cohort Study Site Principal Investigator	UG3/UH3OD023282 (James Gern)
James E.	Gern		MD	University of Wisconsin School of Medicine and Public Health	Madison, Wisconsin, USA	ECHO Cohort Study Site Principal Investigator	UG3/UH3OD023282 (James Gern), UG3OD035509 (Anne Marie Singh)
Diane R.	Gold		MD	Brigham and Women’s Hospital; Harvard Medical School	Boston, Massachusetts, USA	ECHO Cohort Study Site Principal Investigator	UG3/UH3OD023282 (James Gern)
Tina V.	Hartert		MD, MPH	Vanderbilt University Medical Center	Nashville, Tennessee, USA	ECHO Cohort Study Site Principal Investigator	UG3/UH3OD023282 (James Gern), UG3OD035516 and UG3OD035517 (Tina Hartert)

*First name, last name, and suffix (if applicable) are required and will appear in PubMed.

***First Name and Middle Initial(s)**	***Last Name**	***Suffix (eg, Jr, III)**	Academic Degrees	Institution	Location (city, state/province, country)	Role or Contribution, eg, chair, principal investigator	Group (if more than 1 Group listed in the byline) and/or Subgroup (eg, Steering Committee)
Daniel J.	Jackson		MD	University of Wisconsin School of Medicine and Public Health	Madison, Wisconsin, USA	ECHO Cohort Study Site Principal Investigator	UG3/UH3OD023282 (James Gern)
Christine C.	Johnson		PhD	Henry Ford Health	Detroit, Michigan, USA	ECHO Cohort Study Site Principal Investigator	UG3/UH3OD023282 (James Gern), UG3OD035518 (Jennifer Straughen)
Christine L.M.	Joseph		PhD	Henry Ford Health	Detroit, Michigan, USA	ECHO Cohort Study Site Principal Investigator	UG3/UH3OD023282 (James Gern)
Meyer	Kattan		MD	Columbia University Medical Center	New York, New York, USA	ECHO Cohort Study Site Principal Investigator	UG3/UH3OD023282 (James Gern)
Gurjit K.	Khurana Hershey		MD, PhD	Cincinnati Children's Hospital Medical Center	Cincinnati, Ohio, USA	ECHO Cohort Study Site Principal Investigator	UG3/UH3OD023282 (James Gern)
Robert F.	Lemanske, Jr.		MD	University of Wisconsin School of Medicine and Public Health	Madison, Wisconsin, USA	ECHO Cohort Study Site Principal Investigator	UG3/UH3OD023282 (James Gern)
Susan V.	Lynch		PhD	University of California	San Francisco, California, USA	ECHO Cohort Study Site Principal Investigator	UG3/UH3OD023282 (James Gern)
Rachel L.	Miller		MD	Icahn School of Medicine at Mount Sinai	New York, New York, USA	ECHO Cohort Study Site Principal Investigator	UG3/UH3OD023282 (James Gern), UG3/UH3OD023290 (Julie Herbstman)
George T.	O'Connor		MD	Boston University School of Medicine	Boston, Massachusetts, USA	ECHO Cohort Study Site Principal Investigator	UG3/UH3OD023282 (James Gern)

*First name, last name, and suffix (if applicable) are required and will appear in PubMed.

***First Name and Middle Initial(s)**	***Last Name**	***Suffix (eg, Jr, III)**	Academic Degrees	Institution	Location (city, state/province, country)	Role or Contribution, eg, chair, principal investigator	Group (if more than 1 Group listed in the byline) and/or Subgroup (eg, Steering Committee)
Carole	Ober		PhD	University of Chicago	Chicago, Illinois, USA	ECHO Cohort Study Site Principal Investigator	UG3/UH3OD023282 (James Gern), UG3OD035509 (Anne Marie Singh)
Dennis	Ownby		MD	Henry Ford Health	Detroit, Michigan, USA	ECHO Cohort Study Site Principal Investigator	UG3/UH3OD023282 (James Gern)
Katherine	Rivera-Spoljaric		MD	Washington University School of Medicine	St Louis, Missouri, USA	ECHO Cohort Study Site Principal Investigator	UG3/UH3OD023282 (James Gern), UG3OD035521 (Katherine Rivera-Spoljaric)
Patrick H.	Ryan		PhD	University of Cincinnati	Cincinnati, Ohio, USA	ECHO Cohort Study Site Principal Investigator	UG3/UH3OD023282 (James Gern), UG3OD035509 (Anne Marie Singh)
Christine M.	Seroogy		MD	University of Wisconsin School of Medicine and Public Health	Madison, Wisconsin, USA	ECHO Cohort Study Site Principal Investigator	UG3/UH3OD023282 (James Gern)
Anne Marie	Singh		MD	University of Wisconsin School of Medicine and Public Health	Madison, Wisconsin, USA	ECHO Cohort Study Site Principal Investigator	UG3/UH3OD023282 (James Gern), UG3OD035509 (Anne Marie Singh)
Robert A.	Wood		MD	Johns Hopkins University School of Medicine	Baltimore, Maryland, USA	ECHO Cohort Study Site Principal Investigator	UG3/UH3OD023282 (James Gern)
Edward M.	Zoratti		MD	Henry Ford Health	Detroit, Michigan, USA	ECHO Cohort Study Site Principal Investigator	UG3/UH3OD023282 (James Gern), UG3OD035518 (Jennifer Straughen)

*First name, last name, and suffix (if applicable) are required and will appear in PubMed.

***First Name and Middle Initial(s)**	***Last Name**	***Suffix (eg, Jr, III)**	Academic Degrees	Institution	Location (city, state/province, country)	Role or Contribution, eg, chair, principal investigator	Group (if more than 1 Group listed in the byline) and/or Subgroup (eg, Steering Committee)
Rima	Habre		ScD, MSc	University of Southern California	Los Angeles, California, USA	ECHO Cohort Study Site Principal Investigator	UH3OD023287 (Carrie Breton)
Shohreh	Farzan		PhD	University of Southern California	Los Angeles, California, USA	ECHO Cohort Study Site Principal Investigator	UH3OD023287 (Carrie Breton)
Frank D.	Gilliland		MD, MPH, PhD	University of Southern California	Los Angeles, California, USA	ECHO Cohort Study Site Principal Investigator	UH3OD023287 (Carrie Breton)
Irva	Hertz-Picciotto		PhD	University of California, Davis	Davis, California, USA	ECHO Cohort Study Site Principal Investigator	UG3/UH3OD023365 (Irva Hertz-Picciotto), UG3OD035550 (Rebecca Schmidt)
Deborah H.	Bennett		Ph.D	University of California, Davis	Davis, California, USA	ECHO Cohort Study Site Principal Investigator	UG3/UH3OD023365 (Irva Hertz-Picciotto), UG3OD035550 (Rebecca Schmidt)
Julie B.	Schweitzer		Ph.D	University of California, Davis	Davis, California, USA	ECHO Cohort Study Site Principal Investigator	UG3/UH3OD023365 (Irva Hertz-Picciotto)
Rebecca J.	Schmidt		Ph.D	University of California, Davis	Davis, California, USA	ECHO Cohort Study Site Principal Investigator	UG3/UH3OD023365 (Irva Hertz-Picciotto), UG3/UH3OD023342 (Kristen Lyall), UG3OD035550 (Rebecca Schmidt)
Janine M.	LaSalle		PhD	University of California, Davis	Davis, California, USA	ECHO Cohort Study Site Co-Investigator	UG3/UH3OD023365 (Irva Hertz-Picciotto), UG3OD035550 (Rebecca Schmidt)

Supplemental Online Content: Nonauthor Collaborators
*First name, last name, and suffix (if applicable) are required and will appear in PubMed.

***First Name and Middle Initial(s)**	***Last Name**	***Suffix (eg, Jr, III)**	Academic Degrees	Institution	Location (city, state/province, country)	Role or Contribution, eg, chair, principal investigator	Group (if more than 1 Group listed in the byline) and/or Subgroup (eg, Steering Committee)
Alison E.	Hipwell		PhD, ClinPsyD	University of Pittsburgh	Pittsburgh, Pennsylvania, USA	ECHO Cohort Study Site Principal Investigator	UG3/UH3OD023244 (Alison Hipwell)
Kate E.	Keenan		PhD	University of Chicago	Chicago, Illinois, USA	ECHO Cohort Study Site Principal Investigator	UG3/UH3OD023244 (Alison Hipwell)
Catherine J.	Karr		MD, MS, PhD	University of Washington	Seattle, Washington, USA	ECHO Cohort Study Site Principal Investigator	UH3OD023271 and UG3OD035528 (Catherine Karr)
Nicole R.	Bush		PhD	University of California, San Francisco	San Francisco, California, USA	ECHO Cohort Study Site Principal Investigator	UH3OD023271 (Catherine Karr), UG3OD035519 (Qi Zhao)
Kaja Z.	LeWinn		ScD	University of California, San Francisco	San Francisco, California, USA	ECHO Cohort Study Site Principal Investigator	UH3OD023271 (Catherine Karr), UG3OD035519 (Qi Zhao)
Sheela	Sathyanarayana		MD, MPH	University of Washington and Seattle Children's Research Institute	Seattle, Washington, USA	ECHO Cohort Study Site Principal Investigator	UH3OD023271 (Catherine Karr), UG3OD035508 (Sheela Sathyanarayana)
Qi	Zhao		MD, PhD	University of Tennessee Health Science Center	Memphis, Tennessee, USA	ECHO Cohort Study Site Principal Investigator	UH3OD023271 (Catherine Karr), UG3OD035519 (Qi Zhao)
Frances	Tylavsky		DrPH, MS	University of Tennessee Health Science Center	Memphis, Tennessee, USA	ECHO Cohort Study Site Principal Investigator	UH3OD023271 (Catherine Karr)
Kecia N.	Carroll		MD, MPH	Icahn School of Medicine at Mount Sinai	New York, New York, USA	ECHO Cohort Study Site Principal Investigator	UH3OD023271 (Catherine Karr), UG3/UH3OD023337 (Rosalind Wright)

Supplemental Online Content: Nonauthor Collaborators
*First name, last name, and suffix (if applicable) are required and will appear in PubMed.

***First Name and Middle Initial(s)**	***Last Name**	***Suffix (eg, Jr, III)**	Academic Degrees	Institution	Location (city, state/province, country)	Role or Contribution, eg, chair, principal investigator	Group (if more than 1 Group listed in the byline) and/or Subgroup (eg, Steering Committee)
Christine T.	Loftus		MS MPH PhD	University of Washington	Seattle, Washington, USA	ECHO Cohort Study Site Principal Investigator	UH3OD023271 (Catherine Karr)
Leslie D.	Leve		PhD	University of Oregon	Eugene, Oregon, USA	ECHO Cohort Study Site Principal Investigator	UG3/UH3OD023389 (Leslie Leve)
Jody M.	Ganiban		PhD	George Washington University	Washington, DC, USA	ECHO Cohort Study Site Principal Investigator	UG3/UH3OD023389 (Leslie Leve)
Jenae M.	Neiderhiser		PhD	Penn State University	University Park, Pennsylvania, USA	ECHO Cohort Study Site Principal Investigator	UG3/UH3OD023389 (Leslie Leve)
Scott T.	Weiss		MD	Brigham and Women's Hospital and Harvard Medical School	Boston, Massachusetts, USA	ECHO Cohort Study Site Principal Investigator	UH3OD023268 (Scott Weiss)
Augusto A.	Litonjua		MD	Golisano Children's Hospital, University of Rochester	Rochester, New York, USA	ECHO Cohort Study Site Principal Investigator	UH3OD023268 (Scott Weiss)
Cindy T.	McEvoy		MD, MCR	Oregon Health & Science University	Portland, Oregon, USA	ECHO Cohort Study Site Principal Investigator	UG3/UH3OD023288 (Cynthia McEvoy)
Eliot R.	Spindel		MD, PhD	Oregon National Primate Research Center	Beaverton, Oregon, USA	ECHO Cohort Study Site Principal Investigator	UG3/UH3OD023288 (Cynthia McEvoy)
Robert S.	Tepper		MD, PhD	Indiana School of Medicine	Indianapolis, Indiana, USA	ECHO Cohort Study Site Co-Investigator	UG3/UH3OD023288 (Cynthia McEvoy)
Craig J.	Newschaffer		PhD	Penn State	State College, Pennsylvania, USA	ECHO Cohort Study Site Principal Investigator	UG3/UH3OD023342 (Kristen Lyall)

*First name, last name, and suffix (if applicable) are required and will appear in PubMed.

***First Name and Middle Initial(s)**	***Last Name**	***Suffix (eg, Jr, III)**	Academic Degrees	Institution	Location (city, state/province, country)	Role or Contribution, eg, chair, principal investigator	Group (if more than 1 Group listed in the byline) and/or Subgroup (eg, Steering Committee)
Kristen	Lyall		ScD	Drexel University	Philadelphia, Pennsylvania, USA	ECHO Cohort Study Site Principal Investigator	UG3/UH3OD023342 (Kristen Lyall)
Heather E.	Volk		PhD	Johns Hopkins University	Baltimore, Maryland, USA	ECHO Cohort Study Site Principal Investigator	UG3/UH3OD023342 (Kristen Lyall)
Rebecca	Landa		PhD	Center for Autism and Related Disorders, Kennedy Krieger Institute, Johns Hopkins University	Baltimore, Maryland, USA	ECHO Cohort Study Site Co-Investigator	UG3/UH3OD023342 (Kristen Lyall)
Sally	Ozonoff		PhD	University of California Davis	Sacramento, California, USA	ECHO Cohort Study Site Co-Investigator	UG3/UH3OD023342 (Kristen Lyall)
Joseph	Piven		MD	University of North Carolina	Chapel Hill, North Carolina, USA	ECHO Cohort Study Site Co-Investigator	UG3/UH3OD023342 (Kristen Lyall)
Heather	Hazlett		PhD	University of North Carolina	Chapel Hill, North Carolina, USA	ECHO Cohort Study Site Co-Investigator	UG3/UH3OD023342 (Kristen Lyall)
Juhi	Pandey		PhD	Children's Hospital of Philadelphia	Philadelphia, Pennsylvania, USA	ECHO Cohort Study Site Co-Investigator	UG3/UH3OD023342 (Kristen Lyall)
Robert	Schultz		PhD	Children's Hospital of Philadelphia	Philadelphia, Pennsylvania, USA	ECHO Cohort Study Site Co-Investigator	UG3/UH3OD023342 (Kristen Lyall)
Steven	Dager		PhD	University of Washington	Seattle, Washington, USA	ECHO Cohort Study Site Co-Investigator	UG3/UH3OD023342 (Kristen Lyall)
Kelly	Botteron		PhD	Washington University	St Louis, Missouri, USA	ECHO Cohort Study Site Co-Investigator	UG3/UH3OD023342 (Kristen Lyall)
Daniel	Messinger		PhD	University of Miami	Miami, Florida, USA	ECHO Cohort Study Site Co-Investigator	UG3/UH3OD023342 (Kristen Lyall)
Wendy	Stone		PhD	University of Washington	Seattle, Washington, USA	ECHO Cohort Study Site Co-Investigator	UG3/UH3OD023342 (Kristen Lyall)
Jennifer	Ames		PhD	Kaiser Permanente	Oakland, California, USA	ECHO Cohort Study Site Co-Investigator	UG3/UH3OD023342 (Kristen Lyall)

*First name, last name, and suffix (if applicable) are required and will appear in PubMed.

***First Name and Middle Initial(s)**	***Last Name**	***Suffix (eg, Jr, III)**	Academic Degrees	Institution	Location (city, state/province, country)	Role or Contribution, eg, chair, principal investigator	Group (if more than 1 Group listed in the byline) and/or Subgroup (eg, Steering Committee)
Thomas G.	O'Connor		PhD	University of Rochester	Rochester, New York, USA	ECHO Cohort Study Site Principal Investigator	UG3/UH3OD023349 (Thomas O'Connor)
Richard K.	Miller		PhD	University of Rochester	Rochester, New York, USA	ECHO Cohort Study Site Principal Investigator	UG3/UH3OD023349 (Thomas O'Connor)
Emily	Oken		MD, MPH	Harvard Pilgrim Health Care Institute and Harvard Medical School	Boston, Massachusetts, USA	ECHO Cohort Study Site Principal Investigator	UH3OD023286 and UG3OD035533 (Emily Oken)
Michele R.	Hacker		ScD	Beth Israel Deaconess Medical Center	Boston, Massachusetts, USA	ECHO Cohort Study Site Principal Investigator	UG3OD035533 (Emily Oken)
Tamarra	James-Todd		PhD	Harvard Chan School of Public Health	Boston, Massachusetts, USA	ECHO Cohort Study Site Principal Investigator	UG3OD035533 (Emily Oken)
T. Michael	O'Shea	Jr	MD, MPH	University of North Carolina School of Medicine	Chapel Hill, North Carolina, USA	ECHO Cohort Study Site Principal Investigator	UG3/UH3OD023348 (Mike O'Shea), UH3OD023347 (Barry Lester)
Rebecca C.	Fry		PhD	University of North Carolina Gillings School of Global Public Health	Chapel Hill, North Carolina, USA	ECHO Cohort Study Site Principal Investigator	UG3/UH3OD023348 (Mike O'Shea)
Jean A.	Frazier		MD	UMASS Chan Medical School	Worcster, Massachusetts, USA	ECHO Cohort Study Site Co-Investigator	UG3/UH3OD023348 (Mike O'Shea)
Rachana	Singh		MD, MS	Tufts University School of Medicine	Boston, Massachusetts, USA	ECHO Cohort Study Site Co-Investigator	UG3/UH3OD023348 (Mike O'Shea)
Caitlin	Rollins		MD, SM	Harvard Medical School	Boston, Massachusetts, USA	ECHO Cohort Study Site Co-Investigator	UG3/UH3OD023348 (Mike O'Shea)
Angela	Montgomery		MD	Yale School of Medicine	New Haven, Connecticut, USA	ECHO Cohort Study Site Co-Investigator	UG3/UH3OD023348 (Mike O'Shea)

Supplemental Online Content: Nonauthor Collaborators
*First name, last name, and suffix (if applicable) are required and will appear in PubMed.

***First Name and Middle Initial(s)**	***Last Name**	***Suffix (eg, Jr, III)**	Academic Degrees	Institution	Location (city, state/province, country)	Role or Contribution, eg, chair, principal investigator	Group (if more than 1 Group listed in the byline) and/or Subgroup (eg, Steering Committee)
Ruben	Vaidya		MD	University of Massachusetts Chan Medical School-Baystate	Springfield, Massachusetts, USA	ECHO Cohort Study Site Co-Investigator	UG3/UH3OD023348 (Mike O'Shea)
Robert M.	Joseph		PhD	Boston University Chobanian & Avedisian School of Medicine	Boston, Massachusetts, USA	ECHO Cohort Study Site Co-Investigator	UG3/UH3OD023348 (Mike O'Shea)
Lisa K.	Washburn		MD	Wake Forest School of Medicine	Winston-Salem, North Carolina, USA	ECHO Cohort Study Site Co-Investigator	UG3/UH3OD023348 (Mike O'Shea)
Semsa	Gogcu		MD, MPH	Wake Forest School of Medicine; Wake Forest University School of Medicine/Atrium Health Wake Forest	Winston-Salem, North Carolina, USA	ECHO Cohort Study Site Co-Investigator	UG3/UH3OD023348 (Mike O'Shea), UG3OD035513 (Annemarie Stroustrup), UH3OD023320 (Judy Aschner)
Kelly	Bear		DO	ECU Health	Greenville, North Carolina, USA	ECHO Cohort Study Site Co-Investigator	UG3/UH3OD023348 (Mike O'Shea)
Julie V.	Rollins		MA	University of North Carolina School of Medicine	Chapel Hill, North Carolina, USA	ECHO Cohort Study Site Award Project Director	UG3/UH3OD023348 (Mike O'Shea)
Stephen R.	Hooper		PhD	School of Medicine, University of North Carolina at Chapel Hill	Chapel Hill, North Carolina, USA	ECHO Cohort Study Site Co-Investigator	UG3/UH3OD023348 (Mike O'Shea)
Genevieve	Taylor		MD	School of Medicine, University of North Carolina at Chapel Hill	Chapel Hill, North Carolina, USA	ECHO Cohort Study Site Co-Investigator	UG3/UH3OD023348 (Mike O'Shea)
Wesley	Jackson		MD, MPH	University of North Carolina School of Medicine	Chapel Hill, North Carolina, USA	ECHO Cohort Study Site Co-Investigator	UG3/UH3OD023348 (Mike O'Shea)
Amanda	Thompson		PhD	University of North Carolina at Chapel Hill; Gillings School of Global Public Health, University of North Carolina at Chapel Hill	Chapel Hill, North Carolina, USA	ECHO Cohort Study Site Co-Investigator	UG3/UH3OD023348 (Mike O'Shea)
Julie	Daniels		PhD	University of North Carolina at Chapel Hill; Gillings School of Global Public Health, University of North Carolina at Chapel Hill	Chapel Hill, North Carolina, USA	ECHO Cohort Study Site Co-Investigator	UG3/UH3OD023348 (Mike O'Shea)

*First name, last name, and suffix (if applicable) are required and will appear in PubMed.

***First Name and Middle Initial(s)**	***Last Name**	***Suffix (eg, Jr, III)**	Academic Degrees	Institution	Location (city, state/province, country)	Role or Contribution, eg, chair, principal investigator	Group (if more than 1 Group listed in the byline) and/or Subgroup (eg, Steering Committee)
Michelle	Hernandez		MD	School of Medicine, University of North Carolina at Chapel Hill	Chapel Hill, North Carolina, USA	ECHO Cohort Study Site Co-Investigator	UG3/UH3OD023348 (Mike O'Shea)
Kun	Lu		PhD	Gillings School of Global Public Health, University of North Carolina at Chapel Hill	Chapel Hill, North Carolina, USA	ECHO Cohort Study Site Co-Investigator	UG3/UH3OD023348 (Mike O'Shea)
Michael	Msall		MD	University of Chicago Medicine: Comer Children's Hospital	Chicago Illinois, USA	ECHO Cohort Study Site Co-Investigator	UG3/UH3OD023348 (Mike O'Shea)
Madeleine	Lenski		MSPH	Michigan State University	East Lansing, Michigan, USA	ECHO Cohort Study Site Co-Investigator	UG3/UH3OD023348 (Mike O'Shea)
Rawad	Obeid		MD	Beaumont Hospital	Royal Oak, Michigan, USA	ECHO Cohort Study Site Co-Investigator	UG3/UH3OD023348 (Mike O'Shea)
Steven L.	Pastyrnak		PhD	Corewell Health, Helen DeVos Children's Hospital	Grand Rapids, Michigan, USA	ECHO Cohort Study Site Co-Investigator	UG3/UH3OD023348 (Mike O'Shea), UH3OD023347 (Barry Lester)
Elizabeth	Jensen		PhD	Wake Forest University School of Medicine	Winston-Salem, North Carolina, USA	ECHO Cohort Study Site Co-Investigator	UG3/UH3OD023348 (Mike O'Shea)
Christina	Sakai		MD	Mass General Hospital for Children	Boston, Massachusetts, USA	ECHO Cohort Study Site Co-Investigator	UG3/UH3OD023348 (Mike O'Shea)
Hudson	Santos		RN, PhD	University of Miami	Coral Gables, Florida, USA	ECHO Cohort Study Site Principal Investigator	UG3/UH3OD023348 (Mike O'Shea), UG3OD035542 (Hudson Santos)
Jean M.	Kerver		PhD, MSc, RD	Michigan State University, College of Human Medicine	East Lansing, Michigan, USA	ECHO Cohort Study Site Principal Investigator	UG3/UH3OD023285 (Jean Kerver)
Nigel	Paneth		MD, MPH	Michigan State University, College of Human Medicine	East Lansing, Michigan, USA	ECHO Cohort Study Site Principal Investigator	UG3/UH3OD023285 (Jean Kerver)

Supplemental Online Content: Nonauthor Collaborators
*First name, last name, and suffix (if applicable) are required and will appear in PubMed.

***First Name and Middle Initial(s)**	***Last Name**	***Suffix (eg, Jr, III)**	Academic Degrees	Institution	Location (city, state/province, country)	Role or Contribution, eg, chair, principal investigator	Group (if more than 1 Group listed in the byline) and/or Subgroup (eg, Steering Committee)
Charles J.	Barone	II	MD, FAAP	Henry Ford Health	Detroit, Michigan, USA	ECHO Cohort Study Site Principal Investigator	UG3/UH3OD023285 (Jean Kerver), UG3/UH3OD023282 (James Gern)
Michael R.	Elliott		PhD	University of Michigan	Ann Arbor, Michigan, USA	ECHO Cohort Study Site Principal Investigator	UG3/UH3OD023285 (Jean Kerver)
Douglas M.	Ruden		PhD	Wayne State University	Detroit, Michigan, USA	ECHO Cohort Study Site Principal Investigator	UG3/UH3OD023285 (Jean Kerver)
Chris	Fussman		MS	Michigan Department of Health and Human Services (MDHHS)	Lansing, Michigan, USA	ECHO Cohort Study Site Principal Investigator	UG3/UH3OD023285 (Jean Kerver)
Julie B.	Herbstman		PhD	Columbia University Mailman School of Public Health	New York, New York, USA	ECHO Cohort Study Site Principal Investigator	UG3/UH3OD023290 (Julie Herbstman)
Amy	Margolis		PhD	Columbia University Irving Medical Center	New York, New York, USA	ECHO Cohort Study Site Principal Investigator	UG3/UH3OD023290 (Julie Herbstman)
Susan L.	Schantz		PhD	University of Illinois Urbana-Champaign	Urbana, Illinois, USA	ECHO Cohort Study Site Principal Investigator	UG3/UH3OD023272 (Susan Schantz)
Sarah Dee	Geiger		PhD	University of Illinois Urbana-Champaign	Urbana, Illinois, USA	ECHO Cohort Study Site Co-Investigator	UG3/UH3OD023272 (Susan Schantz)
Andrea	Aguiar		PhD	University of Illinois Urbana-Champaign	Urbana, Illinois, USA	ECHO Cohort Study Site Co-Investigator	UG3/UH3OD023272 (Susan Schantz)
Karen	Tabb		PhD, MSW	University of Illinois Urbana-Champaign	Urbana, Illinois, USA	ECHO Cohort Study Site Co-Investigator	UG3/UH3OD023272 (Susan Schantz)
Rita	Strakovsky		PhD	Michigan State University	East Lansing, Michigan, USA	ECHO Cohort Study Site Co-Investigator	UG3/UH3OD023272 (Susan Schantz)

*First name, last name, and suffix (if applicable) are required and will appear in PubMed.

***First Name and Middle Initial(s)**	***Last Name**	***Suffix (eg, Jr, III)**	Academic Degrees	Institution	Location (city, state/province, country)	Role or Contribution, eg, chair, principal investigator	Group (if more than 1 Group listed in the byline) and/or Subgroup (eg, Steering Committee)
Tracey	Woodruff		PhD, MPH	University of California, San Francisco	San Francisco, California, USA	ECHO Cohort Study Site Principal Investigator	UG3/UH3OD023272 (Susan Schantz)
Rachel	Morello-Frosch		PhD, MPH	University of California, Berkeley	Berkeley, California, USA	ECHO Cohort Study Site Principal Investigator	UG3/UH3OD023272 (Susan Schantz)
Amy	Padula		PhD	University of California, San Francisco	San Francisco, California, USA	ECHO Cohort Study Site Co-Investigator	UG3/UH3OD023272 (Susan Schantz)
Joseph B.	Stanford		MD, MSPH	Spencer Fox Eccles School of Medicine, University of Utah	Salt Lake City, Utah, USA	ECHO Cohort Study Site Principal Investigator	UG3/UH3OD023249 (Joseph Stanford)
Christina A.	Porucznik		PhD, MSPH	Spencer Fox Eccles School of Medicine, University of Utah	Salt Lake City, Utah, USA	ECHO Cohort Study Site Principal Investigator	UG3/UH3OD023249 (Joseph Stanford)
Angelo P.	Giardino		MD, PhD	Spencer Fox Eccles School of Medicine, University of Utah	Salt Lake City, Utah, USA	ECHO Cohort Study Site Principal Investigator	UG3/UH3OD023249 (Joseph Stanford)
Rosalind J.	Wright		MD, MPH	Icahn School of Medicine at Mount Sinai	New York, New York, USA	ECHO Cohort Study Site Principal Investigator	UG3/UH3OD023337 (Rosalind Wright)
Robert O.	Wright		MD, MPH	Icahn School of Medicine at Mount Sinai	New York, New York, USA	ECHO Cohort Study Site Principal Investigator	UG3/UH3OD023337 (Rosalind Wright)
Brent	Collett		PhD	University of Washington, Seattle Children's Research Institute	Seattle, Washington, USA	ECHO Cohort Study Site Principal Investigator	UG3OD035508 (Sheela Sathyanarayana)
Nicole	Baumann-Blackmore		MD	University of Wisconsin School of Medicine and Public Health	Madison, Wisconsin, USA	ECHO Cohort Study Site Co-Investigator	UG3OD035509 (Anne Marie Singh)
Ronald	Gangnon		PhD	University of Wisconsin	Madison, Wisconsin, USA	ECHO Cohort Study Site Co-Investigator	UG3OD035509 (Anne Marie Singh)

Supplemental Online Content: Nonauthor Collaborators
*First name, last name, and suffix (if applicable) are required and will appear in PubMed.

*First Name and Middle Initial(s)	*Last Name	*Suffix (eg, Jr, III)	Academic Degrees	Institution	Location (city, state/province, country)	Role or Contribution, eg, chair, principal investigator	Group (if more than 1 Group listed in the byline) and/or Subgroup (eg, Steering Committee)
Daniel J.	Jackson		MD	University of Wisconsin School of Medicine and Public Health	Madison, Wisconsin, USA	ECHO Cohort Study Site Co-Investigator	UG3OD035509 (Anne Marie Singh)
Chris G.	McKennan		PhD	University of Pittsburgh	Pittsburgh, Pennsylvania, USA	ECHO Cohort Study Site Co-Investigator	UG3OD035509 (Anne Marie Singh)
Jo	Wilson		MD	University of Wisconsin School of Medicine and Public Health	Madison, Wisconsin, USA	ECHO Cohort Study Site Co-Investigator	UG3OD035509 (Anne Marie Singh)
Matt	Altman		MD	University of Washington	Seattle, Washington, USA	ECHO Cohort Study Site Co-Investigator	UG3OD035509 (Anne Marie Singh)
Judy L.	Aschner		MD	Albert Einstein College of Medicine; Hackensack Meridian School of Medicine; Center for Discovery and Innovation	Bronx, New York, USA; Nutley, New Jersey, USA	ECHO Cohort Study Site Principal Investigator	UH3OD023320 and UG3OD035546 (Judy Aschner), UG3OD035513 (Annemarie Stroustrup)
Annemarie	Stroustrup		MD, MPH	Northwell Health, Cohen Children's Medical Center, and the Zucker School of Medicine at Hofstra / Northwell	New Hyde Park, New York, USA	ECHO Cohort Study Site Principal Investigator	UH3OD023320 (Judy Aschner), UG3OD035513 (Annemarie Stroustrup)
Stephanie L.	Merhar		MD, MS	Cincinnati Children's	Cincinnati, Ohio, USA	ECHO Cohort Study Site Co-Investigator	UH3OD023320 (Judy Aschner), UG3OD035513 (Annemarie Stroustrup)
Paul E.	Moore		MD	Vanderbilt University Medical Center	Nashville, Tennessee, USA	ECHO Cohort Study Site Co-Investigator	UH3OD023320 (Judy Aschner), UG3OD035513 (Annemarie Stroustrup)
Gloria S.	Pryhuber		MD	University of Rochester Medical Center	Rochester, New York, USA	ECHO Cohort Study Site Co-Investigator	UH3OD023320 (Judy Aschner)
Mark	Hudak		MD	University of Florida College of Medicine	Jacksonville, Florida, USA	ECHO Cohort Study Site Co-Investigator	UH3OD023320 (Judy Aschner)

Supplemental Online Content: Nonauthor Collaborators
*First name, last name, and suffix (if applicable) are required and will appear in PubMed.

***First Name and Middle Initial(s)**	***Last Name**	***Suffix (eg, Jr, III)**	Academic Degrees	Institution	Location (city, state/province, country)	Role or Contribution, eg, chair, principal investigator	Group (if more than 1 Group listed in the byline) and/or Subgroup (eg, Steering Committee)
Ann Marie	Reynolds Lyndaker		MD, MPH	University of Buffalo Jacobs School of Medicine and Biomedical Sciences	Buffalo, New York, USA	ECHO Cohort Study Site Co-Investigator	UH3OD023320 (Judy Aschner)
Andrea L.	Lampland		MD	Children's Minnesota	Minneapolis, Minnesota, USA	ECHO Cohort Study Site Co-Investigator	UH3OD023320 (Judy Aschner)
Burton	Rochelson		MD	Northwell Health and the Zucker School of Medicine at Hofstra / Northwell	New Hyde Park, New York, USA	ECHO Cohort Study Site Principal Investigator	UG3OD035532 (Annemarie Stroustrup)
Sophia	Jan		MD, MSHP	Northwell Health, Cohen Children's Medical Center, and the Zucker School of Medicine at Hofstra / Northwell	New Hyde Park, New York, USA	ECHO Cohort Study Site Co-Investigator	UG3OD035532 (Annemarie Stroustrup)
Matthew J.	Blitz		MD, MBA	Northwell Health and the Zucker School of Medicine at Hofstra / Northwell	New Hyde Park, New York, USA	ECHO Cohort Study Site Co-Investigator	UG3OD035532 (Annemarie Stroustrup)
Michelle W.	Katzow		MD, MS	Northwell Health, Cohen Children's Medical Center, and the Zucker School of Medicine at Hofstra / Northwell	New Hyde Park, New York, USA	ECHO Cohort Study Site Co-Investigator	UG3OD035532 (Annemarie Stroustrup)
Zenobia	Brown		MD, MPH	Northwell Health and the Zucker School of Medicine at Hofstra / Northwell	New Hyde Park, New York, USA	ECHO Cohort Study Site Co-Investigator	UG3OD035532 (Annemarie Stroustrup)
Codruta	Chiuzan		PhD	Northwell Health, Feinstein Institutes for Medical Research	Manhasset, New York, USA	ECHO Cohort Study Site Co-Investigator	UG3OD035532 (Annemarie Stroustrup)
Timothy	Rafael		MD	Northwell Health and the Zucker School of Medicine at Hofstra / Northwell	New Hyde Park, New York, USA	ECHO Cohort Study Site Co-Investigator	UG3OD035532 (Annemarie Stroustrup)
Dawnette	Lewis		MD, MPH	Northwell Health and the Zucker School of Medicine at Hofstra / Northwell	New Hyde Park, New York, USA	ECHO Cohort Study Site Co-Investigator	UG3OD035532 (Annemarie Stroustrup)

*First name, last name, and suffix (if applicable) are required and will appear in PubMed.

***First Name and Middle Initial(s)**	***Last Name**	***Suffix (eg, Jr, III)**	Academic Degrees	Institution	Location (city, state/province, country)	Role or Contribution, eg, chair, principal investigator	Group (if more than 1 Group listed in the byline) and/or Subgroup (eg, Steering Committee)
Natalie	Meirowitz		MD	Northwell Health and the Zucker School of Medicine at Hofstra / Northwell	New Hyde Park, New York, USA	ECHO Cohort Study Site Co-Investigator	UG3OD035532 (Annemarie Stroustrup)
Brenda	Poindexter		MD	Children's Healthcare of Atlanta Emory University	Atlanta, Georgia, USA	ECHO Cohort Study Site Co-Investigator	UH3OD023320 (Judy Aschner)
Tebeb	Gebretsadik		MPH	Vanderbilt University Medical Center	Nashville, Tennessee, USA	ECHO Cohort Study Site Principal Investigator	UG3OD035516 and UG3OD035517 (Tina Hartert)
Sarah	Osmundson		MD, MSC	Vanderbilt University Medical Center	Nashville, Tennessee, USA	ECHO Cohort Study Site Principal Investigator	UG3OD035517 (Tina Hartert)
Jennifer K.	Straughen		PhD	Henry Ford Health	Detroit, Michigan, USA	ECHO Cohort Study Site Principal Investigator	UG3OD035518 (Jennifer Straughen)
Amy	Eapen		MD	Henry Ford Health	Detroit, Michigan, USA	ECHO Cohort Study Site Principal Investigator	UG3OD035518 (Jennifer Straughen)
Andrea	Cassidy-Bushrow		PhD	Henry Ford Health	Detroit, Michigan, USA	ECHO Cohort Study Site Co-Investigator	UG3/UH3OD023282 (James Gern)
Ganesa	Wegienka		PhD	Henry Ford Health	Detroit, Michigan, USA	ECHO Cohort Study Site Co-Investigator	UG3/UH3OD023282 (James Gern)
Alex	Sitarik		MPH	Henry Ford Health	Detroit, Michigan, USA	ECHO Cohort Study Site Biostatistician	UG3/UH3OD023282 (James Gern)
Kim	Woodcroft		PhD	Henry Ford Health	Detroit, Michigan, USA	ECHO Cohort Study Site Co-Investigator	UG3OD035518 (Jennifer Straughen), UG3/UH3OD023282 (James Gern)
Audrey	Urquhart		MPH	Henry Ford Health	Detroit, Michigan, USA	ECHO Cohort Study Site Epidemiologist	UG3OD035518 (Jennifer Straughen), UG3/UH3OD023282 (James Gern)

Supplemental Online Content: Nonauthor Collaborators
*First name, last name, and suffix (if applicable) are required and will appear in PubMed.

***First Name and Middle Initial(s)**	***Last Name**	***Suffix (eg, Jr, III)**	Academic Degrees	Institution	Location (city, state/province, country)	Role or Contribution, eg, chair, principal investigator	Group (if more than 1 Group listed in the byline) and/or Subgroup (eg, Steering Committee)
Albert	Levin		PhD	Henry Ford Health	Detroit, Michigan, USA	ECHO Cohort Study Site Co-Investigator	UG3OD035518 (Jennifer Straughen)
Tisa	Johnson-Hooper		MD	Henry Ford Health	Detroit, Michigan, USA	ECHO Cohort Study Site Co-Investigator	UG3OD035518 (Jennifer Straughen)
Brent	Davidson		MD	Henry Ford Health	Detroit, Michigan, USA	ECHO Cohort Study Site Co-Investigator	UG3/UH3OD023282 (James Gern)
Tengfei	Ma		PhD	Henry Ford Health	Detroit, Michigan, USA	ECHO Cohort Study Site Co-Investigator	UG3OD035518 (Jennifer Straughen)
Emily S.	Barrett		PhD	Environmental and Occupational Health Sciences Institute, Rutgers University	Piscataway, New Jersey, USA	ECHO Cohort Study Site Principal Investigator	UG3OD035527 (Emily S Barrett)
Martin J.	Blaser		MD	Rutgers University	Piscataway, New Jersey, USA	ECHO Cohort Study Site Principal Investigator	UG3OD035527 (Emily S Barrett)
Maria Gloria	Dominguez-Bello		PhD	Rutgers University	New Brunswick, New Jersey, USA	ECHO Cohort Study Site Principal Investigator	UG3OD035527 (Emily S Barrett)
Daniel B.	Horton		MD	Robert Wood Johnson Medical School, Rutgers University	New Brunswick, New Jersey, USA	ECHO Cohort Study Site Principal Investigator	UG3OD035527 (Emily S Barrett)
Manuel	Jimenez		MD	Robert Wood Johnson Medical School, Rutgers University	New Brunswick, New Jersey, USA	ECHO Cohort Study Site Principal Investigator	UG3OD035527 (Emily S Barrett)
Todd	Rosen		MD	Robert Wood Johnson Medical School, Rutgers University	New Brunswick, New Jersey, USA	ECHO Cohort Study Site Co-Investigator	UG3OD035527 (Emily S Barrett)
Kristy	Palomares		MD, PhD	Saint Peter's University Hospital	New Brunswick, New Jersey, USA	ECHO Cohort Study Site Co-Investigator	UG3OD035527 (Emily S Barrett)
Lyndsay A.	Avalos		PhD, MPH	Kaiser Permanente Northern California	Oakland, California, USA	ECHO Cohort Study Site Principal Investigator	UG3OD035540 (Monique Marie Hedderson)

Supplemental Online Content: Nonauthor Collaborators
*First name, last name, and suffix (if applicable) are required and will appear in PubMed.

***First Name and Middle Initial(s)**	***Last Name**	***Suffix (eg, Jr, III)**	Academic Degrees	Institution	Location (city, state/province, country)	Role or Contribution, eg, chair, principal investigator	Group (if more than 1 Group listed in the byline) and/or Subgroup (eg, Steering Committee)
Yeyi	Zhu		PhD, MS	Kaiser Permanente Northern California	Oakland, California, USA	ECHO Cohort Study Site Principal Investigator	UG3OD035540 (Monique Marie Hedderson)
Kelly J .	Hunt		PhD	Medical University of South Carolina	Charleston, South Carolina, USA	ECHO Cohort Study Site Principal Investigator	UG3OD035543 (Kelly J Hunt)
Roger B.	Newman		MD	Medical University of South Carolina	Charleston, South Carolina, USA	ECHO Cohort Study Site Principal Investigator	UG3OD035543 (Kelly J Hunt)
Michael S.	Bloom		PhD	George Mason University	Fairfax, Virginia, USA	ECHO Cohort Study Site Principal Investigator	UG3OD035543 (Kelly J Hunt)
Mallory H.	Alkis		MD	Medical University of South Carolina	Charleston, South Carolina, USA	ECHO Cohort Study Site Co-Investigator	UG3OD035543 (Kelly J Hunt)
James R.	Roberts		MD, MPH	Medical University of South Carolina	Charleston, South Carolina, USA	ECHO Cohort Study Site Co-Investigator	UG3OD035543 (Kelly J Hunt)
Sunni L.	Mumford		PhD	University of Pennsylvania Perelman School of Medicine	Philadelphia, Pennsylvania, USA	ECHO Cohort Study Site Principal Investigator	UG3OD035537 (Sunni L Mumford)
Heather H.	Burris		MD, MPH	Children's Hospital of Philadelphia; University of Pennsylvania Perelman School of Medicine	Philadelphia, Pennsylvania, USA	ECHO Cohort Study Site Principal Investigator	UG3OD035537 (Sunni L Mumford)
Sara B.	DeMauro		MD, MSCE	Children's Hospital of Philadelphia; University of Pennsylvania Perelman School of Medicine	Philadelphia, Pennsylvania, USA	ECHO Cohort Study Site Principal Investigator	UG3OD035537 (Sunni L Mumford)
Lynn M.	Yee		MD, MPH	Feinberg School of Medicine, Northwestern University	Chicago, Illinois, USA	ECHO Cohort Study Site Principal Investigator	UG3OD035546 (Judy Aschner)

*First name, last name, and suffix (if applicable) are required and will appear in PubMed.

***First Name and Middle Initial(s)**	***Last Name**	***Suffix (eg, Jr, III)**	Academic Degrees	Institution	Location (city, state/province, country)	Role or Contribution, eg, chair, principal investigator	Group (if more than 1 Group listed in the byline) and/or Subgroup (eg, Steering Committee)
Aaron	Hamvas		MD	Ann & Robert H. Lurie Children's Hospital, Feinberg School of Medicine, Northwestern University	Chicago, Illinois, USA	ECHO Cohort Study Site Principal Investigator	UG3OD035546 (Judy Aschner)
Antonia F.	Olidipo		MD, MSCI	Hackensack University Medical Center, Hackensack Meridian School of Medicine	Nutley, New Jersey, USA	ECHO Cohort Study Site Co-Investigator	UG3OD035546 (Judy Aschner)
Andrew S.	Haddad		MD	Hackensack University Medical Center, Hackensack Meridian School of Medicine	Nutley, New Jersey, USA	ECHO Cohort Study Site Co-Investigator	UG3OD035546 (Judy Aschner)
Lisa R.	Eiland		MD	Hackensack University Medical Center, Hackensack Meridian School of Medicine	Nutley, New Jersey, USA	ECHO Cohort Study Site Co-Investigator	UG3OD035546 (Judy Aschner)
Nicole T.	Spillane		MD	Hackensack University Medical Center, Hackensack Meridian School of Medicine	Nutley, New Jersey, USA	ECHO Cohort Study Site Co-Investigator	UG3OD035546 (Judy Aschner)
Kirin N.	Suri		MD	Hackensack University Medical Center, Hackensack Meridian School of Medicine	Nutley, New Jersey, USA	ECHO Cohort Study Site Co-Investigator	UG3OD035546 (Judy Aschner)
Stephanie A.	Fisher		MD, MPH	Feinberg School of Medicine, Northwestern University	Chicago, Illinois, USA	ECHO Cohort Study Site Co-Investigator	UG3OD035546 (Judy Aschner)
Jeffrey A.	Goldstein		MD, PhD	Feinberg School of Medicine, Northwestern University	Chicago, Illinois, USA	ECHO Cohort Study Site Co-Investigator	UG3OD035546 (Judy Aschner)
Leena B.	Mithal		MD	Ann & Robert H. Lurie Children's Hospital, Feinberg School of Medicine, Northwestern University	Chicago, Illinois, USA	ECHO Cohort Study Site Co-Investigator	UG3OD035546 (Judy Aschner)
Raye-Ann O.	DeRegnier		MD	Ann & Robert H. Lurie Children's Hospital, Feinberg School of Medicine, Northwestern University	Chicago, Illinois, USA	ECHO Cohort Study Site Co-Investigator	UG3OD035546 (Judy Aschner)

Supplemental Online Content: Nonauthor Collaborators
*First name, last name, and suffix (if applicable) are required and will appear in PubMed.

*First Name and Middle Initial(s)	*Last Name	*Suffix (eg, Jr, III)	Academic Degrees	Institution	Location (city, state/province, country)	Role or Contribution, eg, chair, principal investigator	Group (if more than 1 Group listed in the byline) and/or Subgroup (eg, Steering Committee)
Nathalie L.	Maitre		MD, PhD	Emory University School of Medicine and Cerebral Palsy Foundation	Atlanta, Georgia, USA and New York, New York, USA	ECHO Cohort Study Site Co-Investigator	UG3OD035546 (Judy Aschner)
Ruby H.N.	Nguyen		PhD, MHS	School of Public Health, University of Minnesota	Minneapolis, Minnesota, USA	ECHO award Principal Investigator	UG3OD035529 (Hong-Ngoc Nguyen)
Meghan M.	JaKa		PhD, MS	HealthPartners Institute	Minneapolis, Minnesota, USA	ECHO site Principal Investigator	UG3OD035529 (Hong-Ngoc Nguyen)
Abbey C.	Sidebottom		PhD, MPH	Allina Health	Minneapolis, Minnesota, USA	ECHO site Principal Investigator	UG3OD035529 (Hong-Ngoc Nguyen)
Michael J.	Paidas		MD	University of Miami Miller School of Medicine	Miami, Florida, USA	ECHO site Principal Investigator	UG3OD035542 (Hudson Santos)
JoNell E.	Potter		APRN, PhD	University of Miami Miller School of Medicine	Miami, Florida, USA	ECHO Cohort Study Site Co-Investigator	UG3OD035542 (Hudson Santos)
Natale	Ruby		PhD, PsyD	University of Miami Miller School of Medicine	Miami, Florida, USA	ECHO Cohort Study Site Co-Investigator	UG3OD035542 (Hudson Santos)
Lunthita	Duthely		EdD	University of Miami School of Medicine	Miami, Florida, USA	ECHO Cohort Study Site Co-Investigator	UG3OD035542 (Hudson Santos)
Arumugam	Jayakumar		PhD	University of Miami Miller School of Medicine	Miami, Florida, USA	ECHO Cohort Study Site Co-Investigator	UG3OD035542 (Hudson Santos)
Karen	Young		MD	University of Miami Miller School of Medicine	Miami, Florida, USA	ECHO Cohort Study Site Co-Investigator	UG3OD035542 (Hudson Santos)
Isabel	Maldonado		MPH, BS	University of Miami	Miami, Florida, USA	ECHO Cohort Study Site Program Director	UG3OD035542 (Hudson Santos)
Meghan	Miller		PhD	University of California Davis	Sacramento, California, USA	ECHO Cohort Study Site Co-Investigator	UG3OD035550 (Rebecca Schmidt)
Jonathan L.	Slaughter		MD, MPH	Nationwide Children's Hospital and The Ohio State University	Columbus, Ohio, USA	ECHO Cohort Study Site Principal Investigator	UG3OD035536 (Jonathan Slaughter)

*First name, last name, and suffix (if applicable) are required and will appear in PubMed.

***First Name and Middle Initial(s)**	***Last Name**	***Suffix (eg, Jr, III)**	Academic Degrees	Institution	Location (city, state/province, country)	Role or Contribution, eg, chair, principal investigator	Group (if more than 1 Group listed in the byline) and/or Subgroup (eg, Steering Committee)
Sarah A.	Keim		PhD, MS, MA	Nationwide Children's Hospital and The Ohio State University	Columbus, Ohio, USA	ECHO Cohort Study Site Principal Investigator	UG3OD035536 (Jonathan Slaughter)
Courtney D.	Lynch		PhD, MPH	The Ohio State University	Columbus, Ohio, USA	ECHO Cohort Study Site Principal Investigator	UG3OD035536 (Jonathan Slaughter)
Kartik K.	Venkatesh		MD, PhD	The Ohio State University	Columbus, Ohio, USA	ECHO Cohort Study Site Principal Investigator	UG3OD035536 (Jonathan Slaughter)
Kristina W.	Whitworth		PhD	Baylor College of Medicine	Houston, Texas, USA	ECHO Cohort Study Site Principal Investigator	UG3OD035544 (Kristina Whitworth)
Elaine	Symanski		PhD	Baylor College of Medicine	Houston, Texas, USA	ECHO Cohort Study Site Principal Investigator	UG3OD035544 (Kristina Whitworth)
Thomas F.	Northrup		PhD	University of Texas Health Science Center at Houston (UTHealth Houston) McGovern Medical School	Houston, Texas, USA	ECHO Cohort Study Site Principal Investigator	UG3OD035544 (Kristina Whitworth)
Hector	Mendez-Figueroa		MD	University of Texas Health Science Center at Houston (UTHealth Houston) McGovern Medical School	Houston, Texas, USA	ECHO Cohort Study Site Co-Investigator	UG3OD035544 (Kristina Whitworth)
Ricardo A.	Mosquera		MD	University of Texas Health Science Center at Houston (UTHealth Houston) McGovern Medical School	Houston, Texas, USA	ECHO Cohort Study Site Co-Investigator	UG3OD035544 (Kristina Whitworth)
Margaret R.	Karagas		PhD	Geisel School of Medicine at Dartmouth	Hanover, New Hampshire, USA	ECHO Cohort Study Site Principal Investigator	UG3/UH3OD023275 (Margaret Karagas)

Supplemental Online Content: Nonauthor Collaborators
*First name, last name, and suffix (if applicable) are required and will appear in PubMed.

***First Name and Middle Initial(s)**	***Last Name**	***Suffix (eg, Jr, III)**	Academic Degrees	Institution	Location (city, state/province, country)	Role or Contribution, eg, chair, principal investigator	Group (if more than 1 Group listed in the byline) and/or Subgroup (eg, Steering Committee)
Juliette C.	Madan		MD, MS	Geisel School of Medicine at Dartmouth, Dartmouth Hitchcock Medical Center	Hanover, New Hampshire, USA	ECHO Cohort Study Site Principal Investigator	UG3/UH3OD023275 (Margaret Karagas)
Debra M.	MacKenzie		PhD	College of Pharmacy, University of New Mexico Health Sciences Center	Albuquerque, New Mexico, USA	ECHO Cohort Study Site Principal Investigator	UG3/UH3OD023344 (Debra MacKenzie)
Johnnye L.	Lewis		PhD	College of Pharmacy, University of New Mexico Health Sciences Center	Albuquerque, New Mexico, USA	ECHO Cohort Study Site Principal Investigator	UG3/UH3OD023344 (Debra MacKenzie)
Brandon J.	Rennie		PhD	University of New Mexico	Albuquerque, New Mexico, USA	ECHO Cohort Study Site Co-Investigator	UG3/UH3OD023344 (Debra MacKenzie)
Bennett L.	Leventhal		MD	College of Pharmacy, University of New Mexico Health Sciences Center; University of Chicago	Albuquerque, New Mexico, USA; Chicago, Illinois, USA	ECHO Cohort Study Site Co-Investigator	UG3/UH3OD023344 (Debra MacKenzie)
Young Shin	Kim		MD, MS, MPH, PhD	University of California, San Francisco	San Francisco, California, USA	ECHO Cohort Study Site Co-Investigator	UG3/UH3OD023344 (Debra MacKenzie)
Somer	Bishop		PhD	University of California, San Francisco	San Francisco, California, USA	ECHO Cohort Study Site Co-Investigator	UG3/UH3OD023344 (Debra MacKenzie)
Sara S.	Nozadi		PhD	College of Pharmacy, University of New Mexico Health Sciences Center	Albuquerque, New Mexico, USA	ECHO Cohort Study Site Co-Investigator	UG3/UH3OD023344 (Debra MacKenzie)
Li	Luo		PhD	Comprehensive Cancer Center, University of New Mexico Health Sciences Center	Albuquerque, New Mexico, USA	ECHO Cohort Study Site Co-Investigator	UG3/UH3OD023344 (Debra MacKenzie)
Barry M.	Lester		PhD	Warren Alpert Medical School of Brown University	Providence, Rhode Island, USA	ECHO Cohort Study Site Principal Investigator	UH3OD023347 (Barry Lester)
Carmen J.	Marsit		PhD	Rollins School of Public Health, Emory University	Atlanta, Georgia, USA	ECHO Cohort Study Site Principal Investigator	UH3OD023347 (Barry Lester)

*First name, last name, and suffix (if applicable) are required and will appear in PubMed.

***First Name and Middle Initial(s)**	***Last Name**	***Suffix (eg, Jr, III)**	Academic Degrees	Institution	Location (city, state/province, country)	Role or Contribution, eg, chair, principal investigator	Group (if more than 1 Group listed in the byline) and/or Subgroup (eg, Steering Committee)
Todd	Everson		PhD	Rollins School of Public Health, Emory University	Atlanta, Georgia, USA	ECHO Cohort Study Site Principal Investigator	UH3OD023347 (Barry Lester)
Cynthia M.	Loncar		PhD	Warren Alpert Medical School of Brown University	Providence, Rhode Island, USA	ECHO Cohort Study Site Principal Investigator	UH3OD023347 (Barry Lester)
Elisabeth C.	McGowan		MD	Warren Alpert Medical School of Brown University	Providence, Rhode Island, USA	ECHO Cohort Study Site Principal Investigator	UH3OD023347 (Barry Lester)
Stephen J.	Sheinkopf		PhD	Thompson Center for Autism & Neurodevelopment, University of Missouri	Columbia, Missouri, USA	ECHO Cohort Study Site Principal Investigator	UH3OD023347 (Barry Lester)
Brian S.	Carter		MD	Children's Mercy-Kansas City	Kansas City, Missouri, USA	ECHO Cohort Study Site Principal Investigator	UH3OD023347 (Barry Lester)
Jennifer	Check		MD	Wake Forest School of Medicine	Winston, Salem North Carolina, USA	ECHO Cohort Study Site Principal Investigator	UH3OD023347 (Barry Lester)
Jennifer B.	Helderman		MD	Wake Forest School of Medicine	Winston, Salem North Carolina, USA	ECHO Cohort Study Site Principal Investigator	UH3OD023347 (Barry Lester)
Charles R.	Neal		MD	University of Hawaii John A Burns School of Medicine	Honolulu, Hawaii, USA	ECHO Cohort Study Site Principal Investigator	UH3OD023347 (Barry Lester)
Lynne M.	Smith		MD	UCLA Clinical and Translational Science Institute at The Lundquist Institute, Harbor-UCLA Medical Center	Los Angeles, California, USA	ECHO Cohort Study Site Principal Investigator	UH3OD023347 (Barry Lester)
