# Supplementary material for: Prenatal Diet and Infant Growth From Birth to Age 24 Months
Source: JAMA Netw Open. 2024 Nov 21;7(11):e2445771. doi: 10.1001/jamanetworkopen.2024.45771 (PMC11582932; doi:10.1001/jamanetworkopen.2024.45771)
Supplement: Supplement 3. — Data Sharing Statement [file jamanetwopen-e2445771-s003.pdf]

Data Sharing Statement

Hedderson. Prenatal Diet and Infant Growth From Birth to Age 24 Months. *JAMA Netw Open*. Published November 21, 2024. doi:10.1001/jamanetworkopen.2024.45771

Data
Data available: No
